# Analysis of Homogeneous/Heterogeneous Reactions in an Electrohydrodynamic Environment Utilizing the Second Law

**DOI:** 10.3390/mi14040821

**Published:** 2023-04-06

**Authors:** Farida Aslam, Saima Noreen, Muhammad Idrees Afridi, Muhammad Qasim

**Affiliations:** 1 Department of Mathematics, COMSATS University Islamabad, Park Road Tarlai Kalan, Islamabad 45550, Pakistan; 2 Department of Computing, Abasyn University Islamabad Campus, Islamabad 44000, Pakistan

**Keywords:** Eyring–Powell fluid, electrokinetics, catalyst, second law

## Abstract

In this study, we investigate what happens to entropy in the presence of electrokinetic phenomena. It is speculated that the microchannel has an asymmetrical and slanted configuration. The presence of fluid friction, mixed convection, Joule heating, presence and absence of homogeneity, and a magnetic field are modelled mathematically. It is also emphasized that the diffusion factors of the autocatalyst and the reactants are equal. The governing flow equations are linearized using the Debye–Huckel and lubrication assumptions. The resulting nonlinear couple differential equations are solved using the program’s integrated numerical solver, Mathematica. We take a graphical look at the results of homogeneous and heterogeneous reactions and talk about what we see. It has been demonstrated that homogeneous and heterogeneous reaction parameters affect concentration distribution f in different ways. The Eyring–Powell fluid parameters $B_{1}$ and $B_{2}$ display an opposite relation with the velocity, temperature, entropy generation number, and Bejan number. The mass Grashof number, the Joule heating parameter, and the viscous dissipation parameter all contribute to the overall increase in fluid temperature and entropy.

## 1. Introduction

Homogeneous and heterogeneous chemical reactions are crucial in various fields such as biological processes, catalysis, distillation, combustion, cooling towers, and food processing. Heat and mass transfer in chemical reactions are involved in mechanisms such as evaporation, nuclear reactor cooling, fruit tree groves, polymer development, and pharmaceuticals. Catalysts are employed to accelerate chemical reactions towards the desired reaction limit. Recognizing the importance of homogeneous and heterogeneous reactions, Chaudhary and Merkin [1,2] created a mathematical model to investigate the flow of viscous fluids with homogeneous and heterogeneous reactions that have identical diffusivities in boundary layer flow. The studies [3,4,5] analyze the impact of homogeneous–heterogeneous reactions on fluid flow under different conditions, including nanofluid flow over a porous stretching sheet, micro-polar fluid flow over a permeable stretching or shrinking sheet in a porous medium, and bidirectional nanofluid flow under magnetohydrodynamics with second-order slip velocity.

The complex shear stress–strain rate relationships of non-Newtonian fluids cannot be fully explained by the Navier–Stokes equations. Therefore, several non-Newtonian constitutive models have been developed by scientists to address these complexities. One such model is the Eyring–Powell fluid model [6] introduced by Eyring and Powell in 1944. This model includes elasticity and viscosity. The Eyring–Powell model has an advantage over other non-Newtonian models because its constitutive equations are derived from the kinetic theory of gases rather than the empirical relation in a power law model. At lower and higher shear rates, Eyring–Powell fluid becomes viscous. This model can also explain the shear-thinning characteristics of toothpaste, blood, and ketchup. Existing studies [7,8,9,10] provide valuable insights into the fluid’s behavior at its flow under various conditions—for example, with magnetic fields and heat and mass transfer with mixed convection—and its behavior under chemical reactions, solar radiation, and unsteady squeezing flow. The present article employs the aforementioned fluid model to elucidate the investigation described in the title of this study.

Latham [11] conducted a study on fluid motions in a peristaltic pump for a doctoral dissertation at Massachusetts Institute of Technology. Later, many studies [12,13,14,15,16,17,18,19] focus on the study of peristaltic pumping, a mechanism of fluid transport that mimics the contraction of biological muscles. The studies use various analytical and numerical methods to investigate peristaltic flow, making important contributions to the understanding of this phenomenon and its potential applications.

Pressure-driven flow is limited by frictional losses in miniature devices, but an external electric field can mobilize bulk flow through microfluidic devices. The first mathematical model for viscous fluid flow under the combined effects of electroosmosis and peristalsis was introduced by Chakraborty [20]. In an external electric field, Mallick [21] examined the peristaltic flow of an Eyring–Powell nanofluid. Later, Rafiq and Sajid [22] examined how slip conditions and chemical reaction affect Jeffrey nanofluid peristaltic flow in an electric and magnetic field.

Entropy is produced in combined electro-osmotic peristaltic flows due to the conversion of electrical energy into kinetic energy and the resulting dissipation of energy into heat. Therefore, the efficiency of thermal systems and electrical devices is always affected by the irreversible losses that frequently lead to an increase in entropy and a decrease in thermal efficiency. The generation of entropy has attracted attention in a variety of fields, including the cooling of modern electronic gadgets, heat exchangers, solar power collectors, porous media, combustions, geothermal systems, and turbo machinery. Initially, Bejan [23,24,25] proposed that the rate of entropy creation in convective fluid flow results from heat transfer and viscous shear stress. In Madhu’s study [26], a second law analysis was conducted on an Eyring–Powell fluid under the influence of heat radiation. Alsaedi and Hayat [27] investigated the entropy production in the presence of Ohmic dissipation, mixed convection, and viscous dissipation in MHD Eyring–Powell fluid. In a symmetric channel, Mabood [28] examined the production of entropy caused by peristaltic pumping in the electroosmotic flow of Eyring–Powell fluid.

After a thorough examination of flow mechanisms and the associated effects in the literature mentioned above, it is evident that the generation of entropy in the peristaltic Eyring–Powell flow model under the influence of various factors such as homogeneous and heterogeneous reactions, mixed convection, Joule heating, viscous dissipation, magnetic and electric fields has not been investigated in an inclined asymmetric channel. Therefore, the present study endeavors to fill this research gap in the existing literature. Modeled study can improve heat transfer and reactant mixing in chemical microreactors with peristaltic flows. Entropy produced can also affect the properties of materials synthesized using them. Thus, the study can aid in material development and manufacturing process optimization. Additionally, the study could examine how the Second Law affects the behavior of electrochemical sensors and identify ways to enhance their performance.

## 2. Mathematical Formulation

### 2.1. Geometric Model

**Figure 1:**
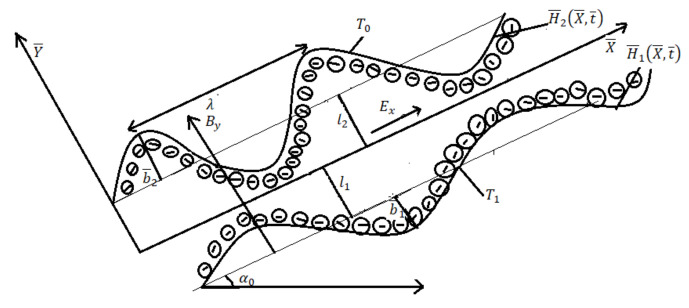
Geometry of the flow problem.

The axial electric field $E_{x}$ and transverse magnetic field $B_{y}$ in Figure 1 influence the incompressible Eyring–Powell fluid’s unsteady electro-osmotic peristaltic flow through a two-dimensional asymmetric channel. The rectangular coordinates $\bar{X}$ and $\bar{Y}$ are taken into account along the flow and perpendicular to flow, respectively. The constant temperature and the potential of the lower and upper walls are represented by $\left(\bar{\zeta_{1}},\;T_{1}\right)$ and $\left(\bar{\zeta_{2}},\;T_{0}\right)$, respectively. The channel is inclined at an angle of $\alpha_{0}$.

Following Merkin and Chaudhary [1], a homogeneous reaction can be written for cubic autocatalysis as

(1) $$A+2B\to 3B,\;\;rate=K_{1}\bar{\overset{´}{a}}{\bar{\overset{´}{b}}}^{2},$$

and the first order heterogeneous reaction associated with a catalyst surface is defined as

(2) $$A\to B,\;\;rate=K_{2}\bar{\overset{´}{a}},$$

in which $\bar{\overset{´}{a}}$ and $\bar{\overset{´}{b}}$ represent the concentrations of chemical species A and B, respectively, and $K_{1}$ and $K_{2}$ denote the rate constants. Further, these two reactions are isothermal. Mathematically, peristaltic asymmetric channel walls are written as:

(3) $$\bar{Y}={\bar{H}}_{1}\left(\bar{X},\;\bar{t}\right)=-l_{1}-{\bar{b}}_{1}\cos(\frac{2\pi }{\lambda }\left(\bar{X}-c\bar{t}\right)+\Phi ),$$

(4) $$\bar{Y}={\bar{H}}_{2}\left(\bar{X},\;\bar{t}\right)=l_{2}+{\bar{b}}_{2}\cos\left(\frac{2\pi }{\lambda }\left(\bar{X}-c\bar{t}\right)\right)\;,$$

where ${\bar{H}}_{1}\left(\bar{X},\bar{t}\right),\;\;{\bar{H}}_{2}\left(\bar{X},\;\bar{t}\right),\;l_{1}+l_{2},\;\lambda ,\;{\bar{b}}_{1},\;{\bar{b}}_{2},\;c,\;\bar{t}\;$, and Φ represent the lower wall, upper wall, channel width, wave length, amplitudes of lower and upper waves, wave speed, time, and phase difference in the laboratory frame. Furthermore, the amplitude, channel width and phase difference satisfy the condition ${\bar{b}}_{1}^{2}+{\bar{b}}_{2}^{2}+2{\bar{b}}_{1}{\bar{b}}_{2}\cos\left(\Phi \right)\leq {\left(l_{1}+l_{2}\right)}^{2}$.

### 2.2. Eyring–Powell Fluid Model

The Cauchy stress tensor ${\bar{\tau }}_{ij}$ for considered fluid is defined as [14,17,18]

(5) $${\bar{\tau }}_{ij}=\mu \bar{\nabla }\;\bar{V}+\frac{1}{\beta^{*}}\sinh^{-1}\left(\frac{1}{c^{*}}\bar{\nabla }\bar{V}\right),$$

where μ is the dynamic viscosity, $\beta^{*}$ and $c^{*}$ are the Eyring–Powell fluid constants, and $\bar{V}$ is the velocity field. Considering

(6) $${sinh}^{-1}\left(\frac{1}{c^{*}}\bar{\nabla }\bar{V}\right)\approx \left(\frac{1}{c^{*}}\bar{\nabla }\bar{V}\right)-\frac{1}{6}{\left(\frac{1}{c^{*}}\bar{\nabla }\bar{V}\right)}^{3},\;\left|\frac{1}{c^{*}}\bar{\nabla }\bar{V}\right|\leq 1,$$

The velocity field $\bar{V}$ having components $\bar{U}$ and $\bar{V}$ along $\bar{X}$ and $\bar{Y}$ is defined as

(7) $$\bar{V}=\left(\bar{U}\left(\bar{X},\;\bar{Y},\;\bar{t}\right),\;\bar{V}\left(\bar{X},\;\bar{Y},\;\bar{t}\right),\;0\right).$$

Using Equations (6) and (7), Equation (5) in component form is written as

(8) $${\bar{\tau }}_{\bar{X}\bar{X}}=\mu \frac{\partial \bar{U}}{\partial \bar{X}}+\frac{1}{\beta^{*}}\left(\frac{1}{c^{*}}\frac{\partial \bar{U}}{\partial \bar{X}}-\frac{1}{6{\left(c^{*}\right)}^{3}}{\left(\frac{\partial \bar{U}}{\partial \bar{X}}\right)}^{3}\right),$$

(9) $${\bar{\tau }}_{\bar{X}\bar{Y}}=\mu \frac{\partial \bar{U}}{\partial \bar{Y}}+\frac{1}{\beta^{*}}\left(\frac{1}{c^{*}}\frac{\partial \bar{U}}{\partial \bar{Y}}-\frac{1}{6{\left(c^{*}\right)}^{3}}{\left(\frac{\partial \bar{U}}{\partial \bar{Y}}\right)}^{3}\right),$$

(10) $${\bar{\tau }}_{\bar{Y}\bar{X}}=\mu \frac{\partial \bar{V}}{\partial \bar{X}}+\frac{1}{\beta^{*}}\left(\frac{1}{c^{*}}\frac{\partial \bar{V}}{\partial \bar{X}}-\frac{1}{6{\left(c^{*}\right)}^{3}}{\left(\frac{\partial \bar{V}}{\partial \bar{X}}\right)}^{3}\right),$$

(11) $${\bar{\tau }}_{\bar{Y}\bar{Y}}=\mu \frac{\partial \bar{V}}{\partial \bar{Y}}+\frac{1}{\beta^{*}}\left(\frac{1}{c^{*}}\frac{\partial \bar{V}}{\partial \bar{Y}}-\frac{1}{6{\left(c^{*}\right)}^{3}}{\left(\frac{\partial \bar{V}}{\partial \bar{Y}}\right)}^{3}\right).$$

### 2.3. Electrical Potential Distribution

The electric potential distribution $\bar{\phi }$ in the EDL of a microchannel is given by the Poisson–Boltzmann equation [20]

(12) $${\bar{\nabla }}^{2}\bar{\phi }=-\frac{\rho_{e}}{\varepsilon \varepsilon_{0}},$$

where $\rho_{e}$ is the total charge density, ε is the relative permittivity of medium, and $\varepsilon_{0}$ is the permittivity of vacuum. For a symmetric electrolyte, the net charge density $\rho_{e}$ obeys Boltzmann distribution and can be defined as [20]

(13) $$\rho_{e}=-ze\left({\bar{\overset{´}{n}}}_{-}-{\bar{\overset{´}{n}}}_{+}\right),$$

where $z,\;e,\;{\bar{\overset{´}{n}}}_{+}$, and ${\bar{\overset{´}{n}}}_{-}$ represent valance of ionic species, charge on proton, cations, and anions, respectively. The charge number distribution of an individual species is defined by the Nernst–Planck equation [20]

(14) $$\left[\frac{\partial {\bar{\overset{´}{n}}}_{\pm }}{\partial \bar{t}}+\bar{U}\frac{\partial {\bar{\overset{´}{n}}}_{\pm }}{\partial \bar{X}}+\bar{V}\frac{\partial {\bar{\overset{´}{n}}}_{\pm }}{\partial \bar{Y}}]=D[\frac{\partial^{2}{\bar{\overset{´}{n}}}_{\pm }}{\partial {\bar{X}}^{2}}+\frac{\partial^{2}{\bar{\overset{´}{n}}}_{\pm }}{\partial {\bar{Y}}^{2}}]\pm \frac{Dze}{T_{av}K_{B}}[\frac{\partial }{\partial \bar{X}}\left({\bar{\overset{´}{n}}}_{\pm }\frac{\partial \bar{\phi }}{\partial \bar{X}}\right)+\frac{\partial }{\partial \bar{Y}}\left({\bar{\overset{´}{n}}}_{\pm }\frac{\partial \bar{\phi }}{\partial \bar{Y}}\right)\right],$$

in which $D,\;T_{av}$ and $K_{B}$ illustrates ionic species diffusivity, $T_{av}$ is the average temperature, and $K_{B}$ is the Boltzmann constant.

We considered the Galilean transformation to shift coordinates and velocities from laboratory frame $\left(\bar{X},\;\bar{Y},\;\bar{t}\right)$ to moving frame $\left(\bar{x},\;\bar{y}\right)$ as

(15) $$\bar{x}=\bar{X}-c\bar{t},\;\bar{y}=\bar{Y},\;\bar{u}=\bar{U}-c,\;\bar{v}=\bar{V},\;{\bar{n}}_{\pm }={\bar{\overset{´}{n}}}_{\pm }.$$

Hence, Equations (3), (4) and (14) become

(16) $$\left[\left(\bar{u}+c\right)\frac{\partial {\bar{n}}_{\pm }}{\partial \bar{x}}+\bar{v}\frac{\partial {\bar{n}}_{\pm }}{\partial \bar{y}}]=D[\frac{\partial^{2}{\bar{n}}_{\pm }}{\partial {\bar{x}}^{2}}+\frac{\partial^{2}{\bar{n}}_{\pm }}{\partial {\bar{y}}^{2}}]\pm \frac{Dze}{T_{av}K_{B}}[\frac{\partial }{\partial \bar{x}}\left({\bar{n}}_{\pm }\frac{\partial \bar{\phi }}{\partial \bar{x}}\right)+\frac{\partial }{\partial \bar{y}}\left({\bar{n}}_{\pm }\frac{\partial \bar{\phi }}{\partial \bar{y}}\right)\right],$$

(17) $${\bar{h}}_{1}\left(\bar{x}\right)=-l_{1}-{\bar{b}}_{1}\cos(\frac{2\pi }{\lambda }\bar{x}+\Phi ),$$

(18) $${\bar{h}}_{2}\left(\bar{x}\right)=l_{2}+{\bar{b}}_{2}\cos\left(\frac{2\pi }{\lambda }\bar{x}\right).$$

This introduces the dimensionless quantities:

(19) $$\phi =\frac{ez}{T_{av}K_{B}}\bar{\phi },\;x=\frac{\bar{x}}{\lambda },\;y=\frac{\bar{y}}{l_{2}},\;b_{1}=\frac{{\bar{b}}_{1}}{l_{2}},\;b_{2}=\frac{{\bar{b}}_{2}}{l_{2}},\;u=\frac{\bar{u}}{c},\;v=\frac{\bar{v}}{c\delta }\;,$$

$$h_{2}=\frac{{\bar{h}}_{2}}{l_{2}},\;n_{\pm }=\frac{{\bar{n}}_{\pm }}{n_{0}},\;\zeta_{1}=\frac{ez}{T_{av}K_{B}}{\bar{\zeta }}_{1},\;\zeta_{2}=\frac{ez}{T_{av}K_{B}}{\bar{\zeta }}_{2},\;\delta =\frac{l_{2}}{\lambda },\;h_{1}=\frac{{\bar{h}}_{1}}{l_{2}},$$

$$m_{e}=\frac{l_{2}}{\lambda_{L}},\;\lambda_{L}=\frac{1}{ez}\sqrt{\frac{\varepsilon \varepsilon_{0}T_{av}K_{B}}{2n_{0}}},\;l=\frac{l_{1}}{l_{2}},\;Pe=\frac{cl_{2}}{D}{,}_{\;}$$

where $\phi ,n_{0},\;\delta ,\;\zeta_{1},\;\zeta_{2},\;l,\;u$, and *v* depict the dimensionless electric potential, bulk ionic concentration, wave number, dimensionless surface potential at the lower and upper walls, semi channel width, and dimensionless velocity components along x and y directions, respectively. Moreover, $m_{e},\;Pe$, and $\lambda_{L}$ are electroosmotic parameter, Peclet number, and Debye length, respectively.

Utilizing Equation (19) in Equation (16), we have

(20) $$Pe\delta \left[\left(u+1\right)\frac{\partial n_{\pm }}{\partial x}+v\frac{\partial n_{\pm }}{\partial y}\right]=\left[\delta^{2}\frac{\partial^{2}n_{\pm }}{\partial x^{2}}+\frac{\partial^{2}n_{\pm }}{\partial y^{2}}\right]\pm [\delta^{2}\frac{\partial }{\partial x}\left(n_{\pm }\frac{\partial \phi }{\partial x}\right),\;+\frac{\partial }{\partial y}\left(n_{\pm }\frac{\partial \phi }{\partial y}\right)].$$

The preceding Equation (20) is simplified by considering a long wavelength and a small ionic Peclet number i-e Pe, δ≪1, yielding

(21) $$\frac{\partial^{2}n_{\pm }}{\partial y^{2}}\pm \frac{\partial }{\partial y}\left(n_{\pm }\frac{\partial \phi }{\partial y}\right)=0.$$

Boundary conditions are:

(22) $$n_{\pm }=1,\;\mathrm{at}\;\phi =0,$$

(23) $$\frac{\partial n_{\pm }}{\partial y}=0,\;\mathrm{at}\;\frac{\partial \phi }{\partial y}=0.$$

Using the above boundary conditions Equation (22) and (23), the general solution of Equation (21) is

(24) $$n_{\pm }=e^{\mp \phi }.$$

Substitute Equation (13) in Equation (12), the Poisson–Boltzmann equation has the form in wave frame

(25) $$\frac{\partial^{2}\bar{\phi }}{\partial {\bar{x}}^{2}}+\frac{\partial^{2}\bar{\phi }}{\partial {\bar{y}}^{2}}=\frac{ze}{\varepsilon \varepsilon_{0}}\left({\bar{n}}_{-}-{\bar{n}}_{+}\right).$$

The dimensional boundary conditions for $\bar{\phi }$ are

$$\bar{\phi }={\bar{\zeta }}_{1},\;\mathrm{at}\;\bar{y}={\bar{h}}_{1}\left(\bar{x}\right),$$

$$\bar{\phi }={\bar{\zeta }}_{2},\;\mathrm{at}\;\bar{y}={\bar{h}}_{2}\left(\bar{x}\right).$$

Using dimensionless variables in Equation (19), the Equations (17), (18) and (25) become

(26) $$\delta^{2}\frac{\partial^{2}\phi }{\partial x^{2}}+\frac{\partial^{2}\phi }{\partial y^{2}}=m_{e}^{2}\sinh\left(\phi \right),$$

$$\phi =\zeta_{1}\;\mathrm{at}\;y=h_{1}\left(x\right)=-l-b_{1}\cos\left(2\pi x+\Phi \right),$$

(27) $$\phi =\zeta_{2}\;\mathrm{at}\;y=h_{2}\left(x\right)=1+b_{2}cos\left(2\pi x\right).$$

Using lubrication [12,13] and Debye–Huckel linearization [20] assumptions, the above Equation (26) simplify to the form

(28) $$\frac{\partial^{2}\phi }{\partial y^{2}}=m_{e}^{2}\phi .$$

Solving Equation (28) with respect to boundary conditions Equation (27), the analytical solution of ϕ is

(29) $$\phi \left(y\right)=\left(\frac{\zeta_{2}\sinh\left(m_{e}h_{1}\right)-\zeta_{1}\sinh\left(m_{e}h_{2}\right)}{\sinh\left(m_{e}h_{1}-m_{e}h_{2}\right)}\right)\cosh\left(m_{e}y\right),\;\;\;\;\;\;\;\;\;\;\;\;\;\;\;\;\;\;\;\;\;\;\;\;\;\;+\left(\frac{\zeta_{1}\cosh\left(m_{e}h_{2}\right)-\zeta_{2}\cosh\left(m_{e}h_{1}\right)}{\sinh\left(m_{e}h_{1}-m_{e}h_{2}\right)}\right)\sinh\left(m_{e}y\right).\;\;\;\;\;\;\;\;\;\;\;\;\;\;\;\;\;\;\;\;\;\;\;\;\;\;\;$$

### 2.4. Flow Analysis

The equations of continuity, momentum, energy, and homogeneous–heterogeneous reaction for Eyring–Powell fluid in the presence of axially applied electric field, inclined channel, transverse magnetic field, and mixed convection in a laboratory frame are [14,16]

(30) $$\frac{\partial \bar{U}}{\partial \bar{X}}+\frac{\partial \bar{V}}{\partial \bar{Y}}=0,$$

(31) $$\rho \left(\frac{\partial \bar{U}}{\partial \bar{t}}+\bar{U}\frac{\partial \bar{U}}{\partial \bar{X}}+\bar{V}\frac{\partial \bar{U}}{\partial \bar{Y}}\right)=-\frac{\partial \bar{P}}{\partial \bar{X}}+\left(\frac{\partial {\bar{\tau }}_{\bar{X}\bar{X}}}{\partial \bar{X}}+\frac{\partial {\bar{\tau }}_{\bar{X}\bar{Y}}}{\partial \bar{Y}}\right)+\rho_{e}E_{x}-\sigma B_{y}^{2}\bar{U}\;+\rho g\beta_{T}\left(\bar{T}-T_{0}\right)sin\;\alpha_{0}+\rho g\beta_{a}\left(\bar{\overset{´}{a}}-a_{0}\right)sin\;\alpha_{0},$$

(32) $$\rho \left(\frac{\partial \bar{V}}{\partial \bar{t}}+\bar{U}\frac{\partial \bar{V}}{\partial \bar{X}}+\bar{V}\frac{\partial \bar{V}}{\partial \bar{Y}}\right)=-\frac{\partial \bar{P}}{\partial \bar{Y}}+\left(\frac{\partial {\bar{\tau }}_{\bar{Y}\bar{X}}}{\partial \bar{X}}+\frac{\partial {\bar{\tau }}_{\bar{Y}\bar{Y}}}{\partial \bar{Y}}\right)+\rho g\beta_{T}\left(\bar{T}-T_{0}\right)cos\;\alpha_{0}\;+\rho g\beta_{a}\left(\bar{\overset{´}{a}}-a_{0}\right)cos\;\alpha_{0},$$

(33) $$\rho c_{p}\left(\frac{\partial \bar{T}}{\partial \bar{t}}+\bar{U}\frac{\partial \bar{T}}{\partial \bar{X}}+\bar{V}\frac{\partial \bar{T}}{\partial \bar{Y}}\right)=k\left(\frac{\partial^{2}\bar{T}}{\partial {\bar{X}}^{2}}+\frac{\partial^{2}\bar{T}}{\partial {\bar{Y}}^{2}}\right)+\sigma E_{x}^{2}+\sigma B_{y}^{2}{\bar{U}}^{2}\;\;\;\;+\left[\frac{\partial \bar{U}}{\partial \bar{X}}{\bar{\tau }}_{\bar{X}\bar{X}}+\frac{\partial \bar{V}}{\partial \bar{Y}}{\bar{\tau }}_{\bar{Y}\bar{Y}}+\left(\frac{\partial \bar{U}}{\partial \bar{Y}}+\frac{\partial \bar{V}}{\partial \bar{X}}\right){\bar{\tau }}_{\bar{X}\bar{Y}}\right],$$

(34) $$\left(\frac{\partial \bar{\overset{´}{a}}}{\partial \bar{t}}+\bar{U}\frac{\partial \bar{\overset{´}{a}}}{\partial \bar{X}}+\bar{V}\frac{\partial \bar{\overset{´}{a}}}{\partial \bar{Y}}\right)=D_{A}\left(\frac{\partial^{2}\bar{\overset{´}{a}}}{\partial {\bar{X}}^{2}}+\frac{\partial^{2}\bar{\overset{´}{a}}}{\partial {\bar{Y}}^{2}}\right)-K_{1}\bar{\overset{´}{a}}{\bar{\overset{´}{b}}}^{2},$$

(35) $$\left(\frac{\partial \bar{\overset{´}{b}}}{\partial \bar{t}}+\bar{U}\frac{\partial \bar{\overset{´}{b}}}{\partial \bar{X}}+\bar{V}\frac{\partial \bar{\overset{´}{b}}}{\partial \bar{Y}}\right)=D_{B}\left(\frac{\partial^{2}\bar{\overset{´}{b}}}{\partial {\bar{X}}^{2}}+\frac{\partial^{2}\bar{\overset{´}{b}}}{\partial {\bar{Y}}^{2}}\right)+K_{1}\bar{\overset{´}{a}}{\bar{\overset{´}{b}}}^{2}.$$

$\beta_{T}$ is the thermal expansion coefficient, $\beta_{a}$ is the concentration expansion coefficient, $a_{0}$ is the uniform concentration of reactant A, $\alpha_{0}$ is the angle of inclination,$\;c_{p}$ is the specific heat, $D_{A}$ and $D_{B}$ are the coefficients of mass diffusivity for homogeneous and heterogeneous reactions.

The stationary frame and moving frame are related by the following transformations:

(36) $$\bar{x}=\bar{X}-c\bar{t},\;\bar{y}=\bar{Y},\;\bar{u}\left(\bar{x},\;\bar{y}\right)=\bar{U}\left(\bar{X},\;\bar{Y},\;\bar{t}\right)-c\bar{t},\bar{v}\left(\bar{x},\;\bar{y}\right)=\bar{V}\left(\bar{X},\;\bar{Y},\;\bar{t}\right),$$

$$T\left(\bar{x},\bar{y}\right)=\bar{T}\left(\bar{X},\;\bar{Y},\;\bar{t}\right),\;\bar{a}\left(\bar{x},\;\bar{y}\right)=\bar{\overset{´}{a}}\left(\bar{X},\;\bar{Y},\;\bar{t}\right),\;\bar{b}\left(\bar{x},\;\bar{y}\right)=\bar{\overset{´}{b}}\left(\bar{X},\;\bar{Y},\;\bar{t}\right),$$

$$\bar{p}\left(\bar{x},\bar{y}\right)=\bar{P}\left(\bar{X},\;\bar{Y},\;\bar{t}\right).$$

By employing the above transformations Equation (36), Equations (30)–(35) yield

(37) $$\frac{\partial \bar{u}}{\partial \bar{x}}+\frac{\partial \bar{v}}{\partial \bar{y}}=0,$$

(38) $$\rho \left(\left(\bar{u}+c\right)\frac{\partial \bar{u}}{\partial \bar{x}}+\bar{v}\frac{\partial \bar{u}}{\partial \bar{y}}\right)=-\frac{\partial \bar{p}}{\partial \bar{x}}+\left(\frac{\partial {\bar{\tau }}_{\bar{x}\bar{x}}}{\partial \bar{x}}+\frac{\partial {\bar{\tau }}_{\bar{x}\bar{y}}}{\partial \bar{y}}\right)+\rho g\beta_{T}\left(T-T_{0}\right)sin\;\alpha_{0}\;+\rho g\beta_{a}\left(\bar{a}-a_{0}\right)sin\;\alpha_{0}-\varepsilon \varepsilon_{0}E_{x}\left(\frac{\partial^{2}\bar{\phi }}{\partial {\bar{x}}^{2}}+\frac{\partial^{2}\bar{\phi }}{\partial {\bar{y}}^{2}}\right)+\sigma B_{y}^{2}\left(\bar{u}+c\right),$$

(39) $$\rho \left(\left(\bar{u}+c\right)\frac{\partial \bar{v}}{\partial \bar{x}}+\bar{v}\frac{\partial \bar{v}}{\partial \bar{y}}\right)=-\frac{\partial \bar{p}}{\partial \bar{y}}+\left(\frac{\partial {\bar{\tau }}_{\bar{y}\bar{x}}}{\partial \bar{x}}+\frac{\partial {\bar{\tau }}_{\bar{y}\bar{y}}}{\partial \bar{y}}\right)+\rho g\beta_{T}\left(T-T_{0}\right)\;cos\;\alpha_{0}\;+\rho g\beta_{a}\left(\bar{a}-a_{0}\right)cos\;\alpha_{0},$$

(40) $$\rho c_{p}\left(\left(\bar{u}+c\right)\frac{\partial T}{\partial \bar{x}}+\bar{v}\frac{\partial T}{\partial \bar{y}}\right)=k\left(\frac{\partial^{2}T}{\partial {\bar{x}}^{2}}+\frac{\partial^{2}T}{\partial {\bar{y}}^{2}}\right)+\left[\frac{\partial \bar{u}}{\partial \bar{x}}{\bar{\tau }}_{\bar{x}\bar{x}}+\frac{\partial \bar{v}}{\partial \bar{y}}{\bar{\tau }}_{\bar{y}\bar{y}}+\left(\frac{\partial \bar{u}}{\partial \bar{y}}+\frac{\partial \bar{v}}{\partial \bar{x}}\right){\bar{\tau }}_{\bar{x}\bar{y}}\right]\;+\sigma E_{x}^{2}+\sigma B_{y}^{2}{\left(\bar{u}+c\right)}^{2},$$

(41) $$\left(\left(\bar{u}+c\right)\frac{\partial \bar{a}}{\partial \bar{x}}+\bar{v}\frac{\partial \bar{a}}{\partial \bar{y}}\right)=D_{A}\left(\frac{\partial^{2}\bar{a}}{\partial {\bar{x}}^{2}}+\frac{\partial^{2}\bar{a}}{\partial {\bar{y}}^{2}}\right)-K_{1}\bar{a}{\bar{b}}^{2},$$

(42) $$\left(\left(\bar{u}+c\right)\frac{\partial \bar{b}}{\partial \bar{x}}+\bar{v}\frac{\partial \bar{b}}{\partial \bar{y}}\right)=D_{B}\left(\frac{\partial^{2}\bar{b}}{\partial {\bar{x}}^{2}}+\frac{\partial^{2}\bar{b}}{\partial {\bar{y}}^{2}}\right)+K_{1}\bar{a}{\bar{b}}^{2}.$$

Let us now introduce the non-dimensional parameters listed below as:

(43) $$p=\frac{l_{2}^{2}}{\lambda c\mu }\bar{p},\;\tau =\frac{l_{2}}{c\mu }\bar{\tau },\;\theta =\frac{T-T_{0}}{T_{1}-T_{0}}\;,\;u_{HS}=-\frac{\varepsilon \varepsilon_{0}E_{x}T_{av}K_{B}}{ez\mu },\;U_{HS}=\frac{u_{HS}}{c},$$

$$Pr=\frac{c_{p}\mu }{k}\;,\;Br=\frac{c^{2}\mu }{k\left(T_{1}-T_{0}\right)}\;,\;Ha=B_{y}l_{2}\sqrt{\frac{\sigma }{\mu }}\;,\;\gamma =\frac{\sigma E_{x}^{2}l_{2}^{2}}{k\left(T_{1}-T_{0}\right)},\;M_{2}=\frac{K_{2}l_{2}}{D_{A}},$$

$$\overset{´}{f}=\frac{\bar{a}-a_{0}}{a_{0}}\;,\;f=\overset{´}{f}+1,\;\overset{´}{g}=\frac{\bar{b}}{a_{0}}\;,\;G_{t}=\frac{\rho gl_{2}^{2}\beta_{T}\left(T_{1}-T_{0}\right)}{\mu c}\;,\;G_{a}=\frac{\rho gl_{2}^{2}\beta_{a}a_{0}}{\mu c}\;,$$

$$M_{1}=\frac{\rho K_{1}a_{0}^{2}l_{2}^{2}}{\mu },\;B_{1}=\frac{1}{\mu \beta^{*}c^{*}},\;B_{2}=\frac{B_{1}c^{2}}{l_{2}^{2}{\left(c^{*}\right)}^{2}},\;Re=\frac{\rho cl_{2}}{\mu },\;Sc=\frac{\mu }{\rho D_{A}},\;D_{R}=\frac{D_{B}}{D_{A}},$$

where $\theta ,\;Ha,\;u_{HS,}\;U_{HS},\;Re,\;\gamma ,\;Sc,\;Pr,\;Br,\;D_{R},\;f,\;\overset{´}{g},\;G_{t},\;G_{a},\;M_{1},\;M_{2},\;B_{1}$, and $B_{2}$ are the dimensionless temperature, Hartmann number, Helmholtz–Smoluchowski velocity, the ratio of the Helmholtz–Smoluchowski velocity to the characteristic wave speed, Reynolds number, Joule heating parameter, Schmidt number, Prandtl number, Brinkman number, diffusion ratio coefficient, dimensionless concentration of species A, dimensionless concentration of species B, temperature Grashoff number, concentration Grashoff number, homogeneous reaction parameter, heterogeneous reaction parameter, and Eyring–Powell fluid parameters, respectively.

The dimensionless boundary conditions are [16]

$$u=-1,\;\theta =1,\;\frac{\partial f}{\partial y}-M_{2}f=0,\;D_{R}\frac{\partial \overset{´}{g}}{\partial y}+M_{2}\overset{´}{g}=0\;\mathrm{at}\;y=h_{1},$$

(44) $$u=-1,\;\theta =0,\;\frac{\partial f}{\partial y}-M_{2}f=0,\;D_{R}\frac{\partial \overset{´}{g}}{\partial y}+M_{2}\overset{´}{g}=0\;\mathrm{at}\;y=h_{2}.$$

It is assumed that $D_{A}=D_{B}$ i-e $D_{R}=1$. This assumption results in the given relation [16]

(45) $$f+\overset{´}{g}=1.$$

From the assumption of a low Reynolds number and a long wavelength [12,13], we have the following system of equations

(46) $$\frac{\partial u}{\partial x}+\frac{\partial v}{\partial y}=0,$$

(47) $$\frac{\partial p}{\partial x}=\left(\frac{\partial \tau_{xy}}{\partial y}\right)+U_{HS}m_{e}^{2}\phi -{\left(Ha\right)}^{2}\left(u+1\right)+G_{t}\theta \;sin\;\alpha_{0}+G_{a}f\;sin\;\alpha_{0},$$

(48) $$\frac{\partial p}{\partial y}=0,$$

(49) $$\frac{\partial^{2}\theta }{\partial y^{2}}+Br\frac{\partial u}{\partial y}\tau_{xy}+\gamma +{\left(Ha\right)}^{2}Br{\left(u+1\right)}^{2}=0,$$

(50) $$\frac{1}{Sc}\frac{\partial^{2}f}{\partial y^{2}}-M_{1}f\left(1-f^{2}\right)=0,$$

(51) $$\tau_{xy}=\left(1+B_{1}\right)\frac{\partial u}{\partial y}-B_{2}{\left(\frac{\partial u}{\partial y}\right)}^{3}.$$

Eliminating the pressure term from Equations (47) and (48), we obtain

(52) $$\left(1+B_{1}\right)\frac{\partial^{3}u}{\partial y^{3}}-2B_{2}\frac{\partial u}{\partial y}{\left(\frac{\partial^{2}u}{\partial y^{2}}\right)}^{2}-B_{2}{\left(\frac{\partial u}{\partial y}\right)}^{2}\frac{\partial^{3}u}{\partial y^{3}}+U_{HS}m_{e}^{2}\frac{\partial \phi }{\partial y}-{\left(Ha\right)}^{2}\frac{\partial u}{\partial y}\;+G_{t}\frac{\partial \theta }{\partial y}sin\;\alpha_{0}+G_{a}\frac{\partial f}{\partial y}sin\;\alpha_{0}=0.$$

Introducing the stream function ψ with velocity components $u=\frac{\partial \psi }{\partial y}$ and $v=-\frac{\partial \psi }{\partial x}$, Equation (46) is automatically satisfied and Equations (49), (50) and (52) become

(53) $$\left(1+B_{1}\right)\frac{\partial^{4}\psi }{\partial y^{4}}-2B_{2}\frac{\partial^{2}\psi }{\partial y^{2}}{\left(\frac{\partial^{3}\psi }{\partial y^{3}}\right)}^{2}-B_{2}{\left(\frac{\partial^{2}\psi }{\partial y^{2}}\right)}^{2}\frac{\partial^{4}\psi }{\partial y^{4}}+U_{HS}m_{e}^{2}\frac{\partial \phi }{\partial y}\;-{\left(Ha\right)}^{2}\frac{\partial^{2}\psi }{\partial y^{2}}+G_{t}\frac{\partial \theta }{\partial y}sin\;\alpha_{0}+G_{a}\frac{\partial f}{\partial y}sin\;\alpha_{0}=0,$$

(54) $$\frac{\partial^{2}\theta }{\partial y^{2}}+Br\frac{\partial^{2}\psi }{\partial y^{2}}\left(\left(1+B_{1}\right)\frac{\partial^{2}\psi }{\partial y^{2}}-\frac{B_{2}}{3}{\left(\frac{\partial^{2}\psi }{\partial y^{2}}\right)}^{3}\right)+\gamma +{\left(Ha\right)}^{2}Br{\left(\frac{\partial \psi }{\partial y}+1\right)}^{2}=0,$$

(55) $$\frac{1}{Sc}\frac{\partial^{2}f}{\partial y^{2}}-M_{1}f\left(1-f^{2}\right)=0.$$

The boundary conditions are:

$$\psi =-\frac{F}{2},\;\frac{\partial \psi }{\partial y}=-1,\;\theta =1,\;\frac{\partial f}{\partial y}-M_{2}f=0\;\mathrm{at}\;y=h_{1}\left(x\right),$$

(56) $$\psi =\frac{F}{2},\;\frac{\partial \psi }{\partial y}=-1,\;\theta =0,\;f=1\;\mathrm{at}\;y=h_{2}\left(x\right),$$

where

(57) $$F=\overset{h_{2}\left(x\right)}{\underset{h_{1}\left(x\right)}{\int }}\frac{\partial \psi }{\partial y}dy=\psi \left[h_{2}\left(x\right)]-\psi [h_{1}\left(x\right)\right]$$

is related to Θ by

(58) Θ=F+1+l.

The dimensionless pressure rise per wavelength Δp is written as:

(59) $$\Delta p=\int_{0}^{1}\frac{\partial p}{\partial x}dx.$$

## 3. Entropy Generation

The development of entropy is a consequence of the irreversibility of heat transfer in thermodynamic processes. Rate of local entropy creation per unit volume for an incompressible Eyring–Powell fluid, subject to the effects of viscous dissipation, electric field, heat transfer, and magnetic fields, can be written as:

(60) $${\bar{En}}_{gen}=\frac{k}{T^{2}}\left[{\left(\frac{\partial T}{\partial \bar{x}}\right)}^{2}+{\left(\frac{\partial T}{\partial \bar{y}}\right)}^{2}\right]+\frac{1}{T}\left[\frac{\partial \bar{u}}{\partial \bar{x}}{\bar{\tau }}_{\bar{x}\bar{x}}+\frac{\partial \bar{v}}{\partial \bar{y}}{\bar{\tau }}_{\bar{y}\bar{y}}+\left(\frac{\partial \bar{u}}{\partial \bar{y}}+\frac{\partial \bar{v}}{\partial \bar{x}}\right){\bar{\tau }}_{\bar{x}\bar{y}}\right]\;+\frac{\sigma E_{x}^{2}}{T}+\frac{\sigma B_{y}^{2}}{T}{\left(\bar{u}+c\right)}^{2}.$$

The entropy generation number $N_{s}$, which is the ratio of volumetric entropy generation rate to the characteristic entropy generation $(En_{gen}=k/l_{2}^{2}$), is given as

(61) $$N_{s}=\frac{{\bar{En}}_{gen}}{En_{gen}}=\frac{1}{{\left(\theta +\varrho \right)}^{2}}{\left(\frac{\partial \theta }{\partial y}\right)}^{2}+\frac{Br}{\left(\theta +\varrho \right)}\left[\left(1+B_{1}\right){\left(\frac{\partial^{2}\psi }{\partial y^{2}}\right)}^{2}-\frac{B_{2}}{3}{\left(\frac{\partial^{2}\psi }{\partial y^{2}}\right)}^{4}\right]\;+\frac{\gamma }{\left(\theta +\varrho \right)}+\frac{{\left(Ha\right)}^{2}Br}{\left(\theta +\varrho \right)}{\left(\frac{\partial \psi }{\partial y}+1\right)}^{2},$$

where $\varrho =\frac{T_{0}}{T_{1}-T_{0}}$ is the temperature difference parameter.

The Bejan number Be is the ratio of heat transfer irreversibility to the total entropy generation. From a mathematical perspective, it is defined as

(62) $$Be=\frac{\frac{1}{{\left(\theta +\varrho \right)}^{2}}{\left(\frac{\partial \theta }{\partial y}\right)}^{2}}{\begin{array}{c} {}[\frac{1}{{\left(\theta +\varrho \right)}^{2}}{\left(\frac{\partial \theta }{\partial y}\right)}^{2}+\frac{Br}{\left(\theta +\varrho \right)}\left[\left(1+B_{1}\right){\left(\frac{\partial^{2}\psi }{\partial y^{2}}\right)}^{2}-\frac{B_{2}}{3}{\left(\frac{\partial^{2}\psi }{\partial y^{2}}\right)}^{4}\right]+\frac{\gamma }{\left(\theta +\varrho \right)}\\ +\frac{{\left(Ha\right)}^{2}Br}{\left(\theta +\varrho \right)}{\left(\frac{\partial \psi }{\partial y}+1\right)}^{2}] \end{array}}$$

The Bejan number can range from 0 to 1. Hence, a value of Be that is equal to one implies that thermal irreversibility is predominant, whereas a value of Be that is equal to zero shows that entropy formation as a result of viscous dissipation, magnetic field, and Joule dissipation is predominant. Be=0.5 indicates that irreversibility in the generation of entropy due to heat transmission is equivalent to irreversibility in the generation of entropy due to fluid friction, electric field, and magnetic field.

## 4. Solution of the Problem

Mathematica is an important tool for numerically solving partial differential equations. It provides versatility, precision, visualisation, integration with other Mathematica functions, and efficiency, making it a potent tool for a variety of applications in science, engineering, and mathematics. Such a methodology has the advantage of automatically choosing the best algorithm and tracking error within the given minimum and maximum range. Therefore, the differential Equations (53)–(55) are coupled nonlinear even after invoking low Reynolds number and long wavelength approximation. It is challenging to explicitly solve such a system and obtain the exact solution. Thus, using the built-in tool in the Mathematica software, the numerical approximations of Equations (53)–(55) with boundary conditions (56) are made for the velocity, temperature, and concentration distributions. 

## 5. Computational Results and Discussion

**Figure 2:**
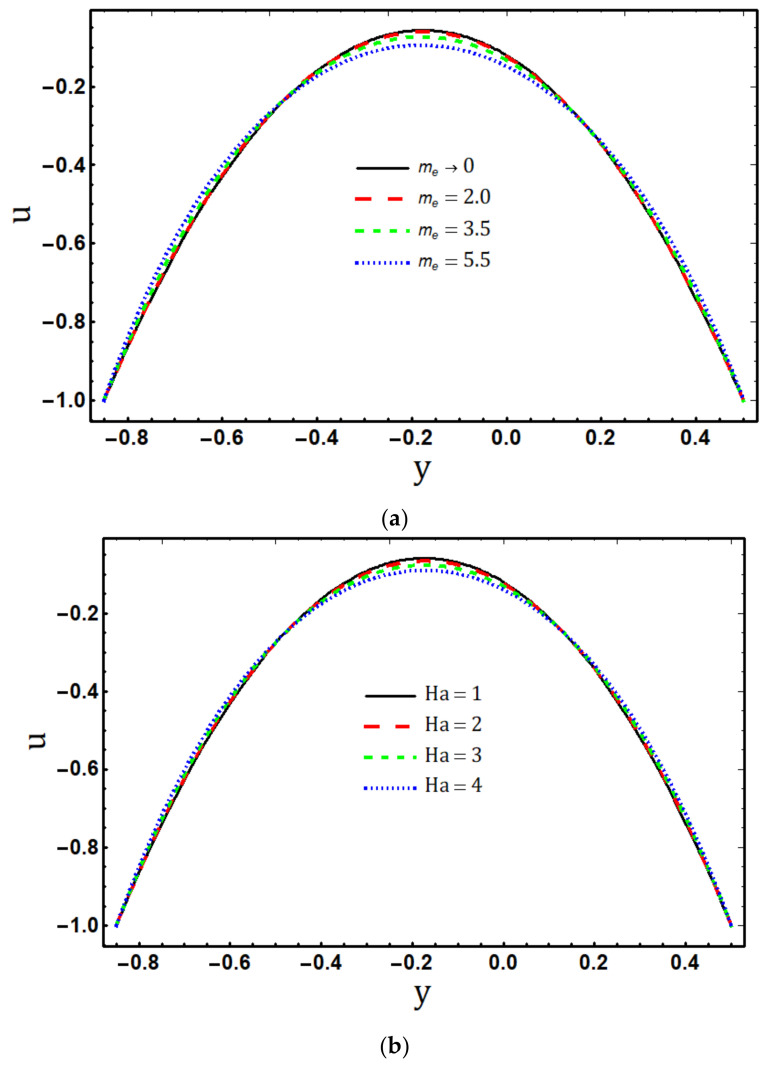

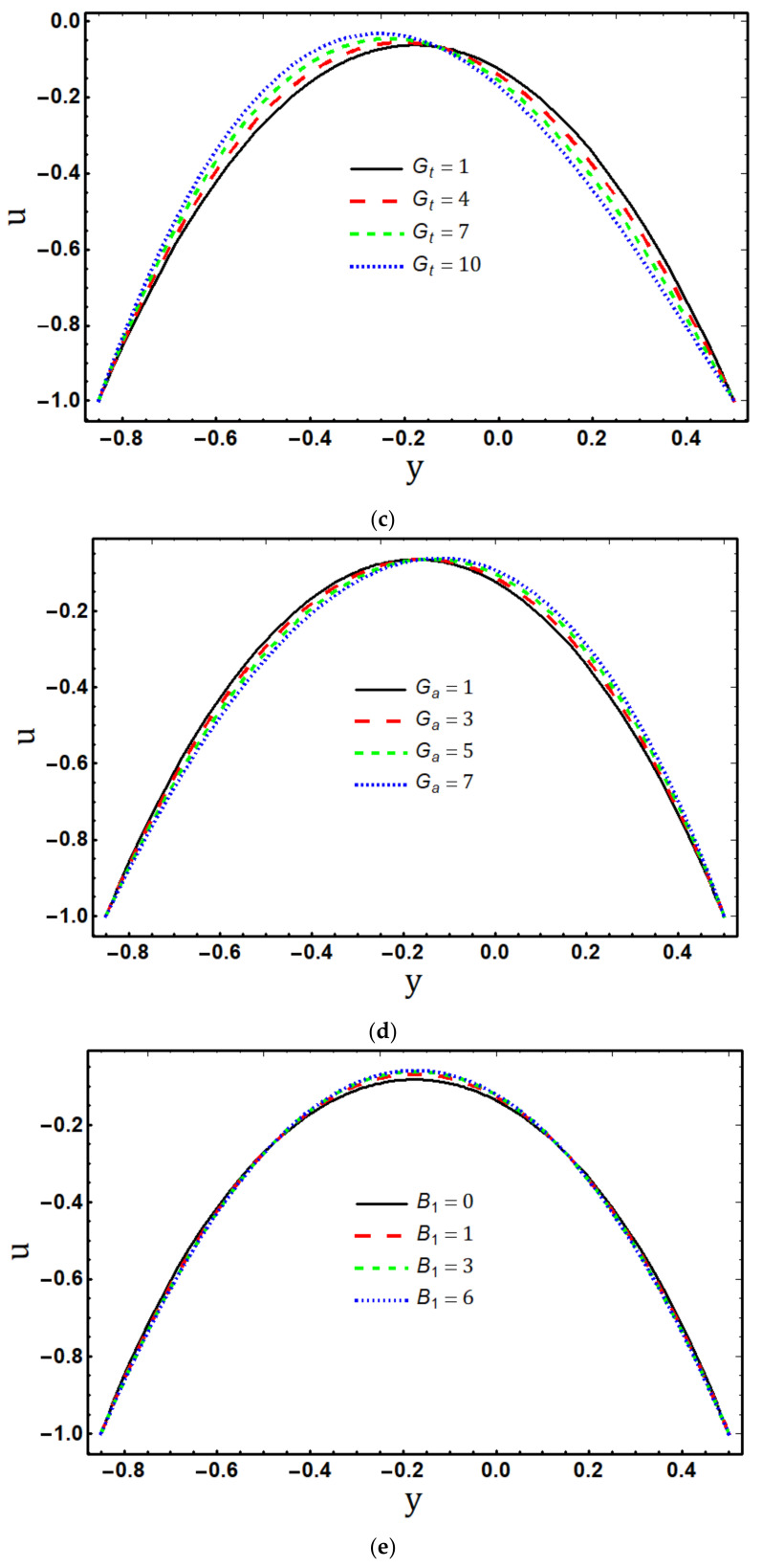

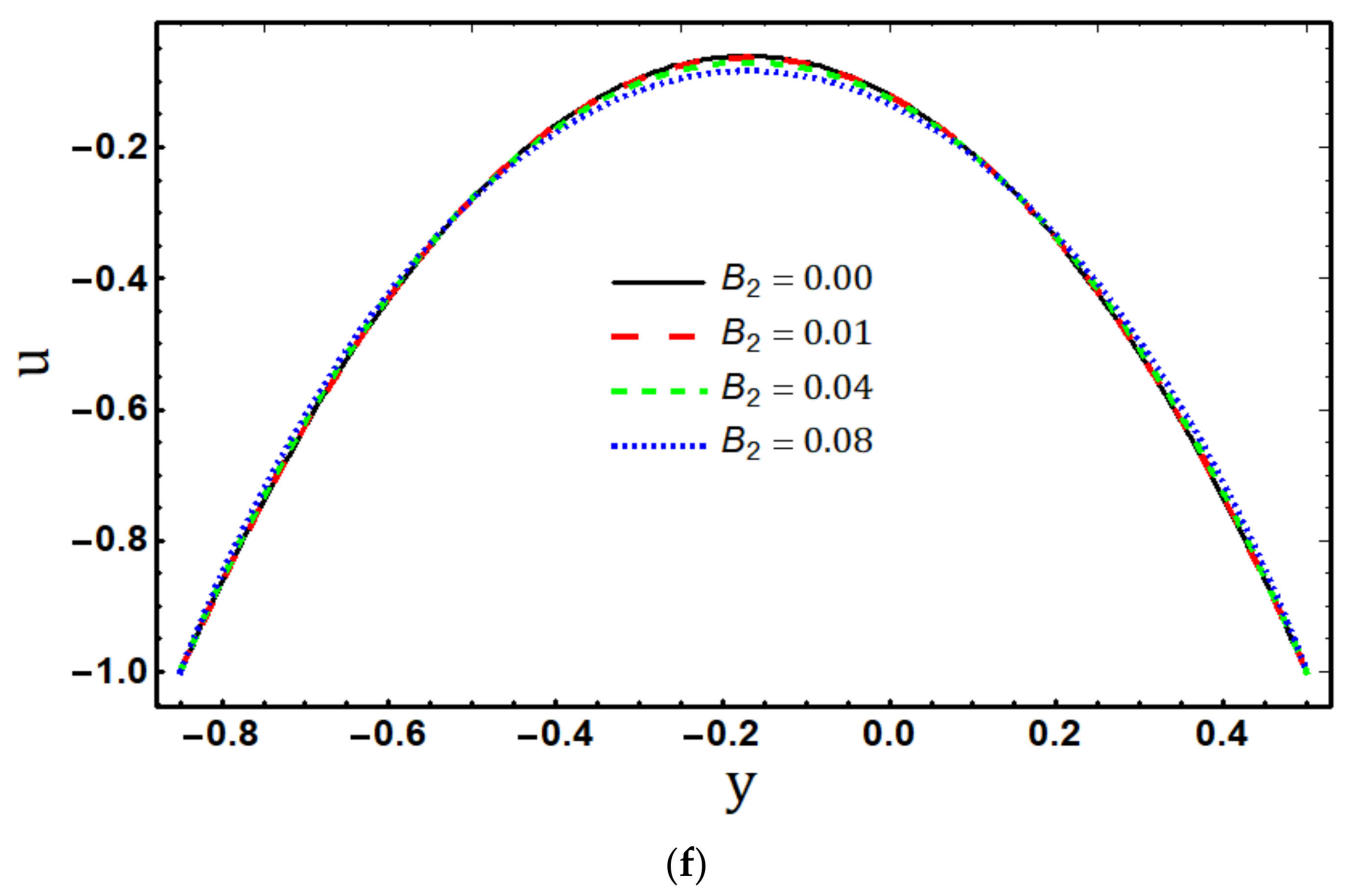
(**a**): Effects of $m_{e}$ on u, while other parameters are $b_{1}=0.3,\;b_{2}=0.5,\;x=0.5,\;l=1,$ $\zeta_{1}=-0.9,\;\zeta_{2}=-0.8,\;U_{HS}=-0.9,\;\Theta =1.5,\;\Phi =\pi /3,\;\alpha =\;\pi /3,\;Ha=0.1,\;B_{1}=2,\;M_{1}=1,$ $B_{2}=0.02,\;G_{t}=0.6,\;G_{a}=0.5,\;Sc=1.5,\;M_{2}=2,\;\gamma =0.6,\;Br=0.5$. (**b**): Effects of Ha on u, while other parameters are $b_{1}=0.3,\;b_{2}=0.5,\;x=0.5,\;l=1,$ $\zeta_{1}=-0.5,\;\zeta_{2}=-0.4,\;U_{HS}=-0.75,\;\Theta =1.5,\;\Phi =\pi /3,\;\alpha =\;\pi /3,\;m_{e}=3,\;B_{1}=2,\;M_{1}=1$, $B_{2}=0.02,\;G_{t}=0.6,\;G_{a}=0.5,\;Sc=1.5,\;M_{2}=1.5,\;\gamma =0.6,\;Br=0.5$. (**c**): Effects of $G_{t}$ on u, while other parameters are $b_{1}=0.3,\;b_{2}=0.5,\;x=0.5,\;l=1,\zeta_{1}=-0.5,\;\zeta_{2}=-0.4,\;U_{HS}=-0.5,\;\Theta =1.5,\;\Phi =\pi /3,\;\alpha =\;\pi /3,\;Ha=1,\;B_{1}=0.3,\;M_{1}=1.5$, $B_{2}=0.02,\;m_{e}=1,\;G_{a}=0.8,\;Sc=1.5,\;M_{2}=3,\;\gamma =0.6,\;Br=0.5$. (**d**): Effects of $G_{a}$ on u, while other parameters are $b_{1}=0.3,\;b_{2}=0.5,\;x=0.5,\;l=1,\zeta_{1}=-0.5,\;\zeta_{2}=-0.4,\;U_{HS}=-0.5,\;\Theta =1.5,\;\Phi =\pi /3,\;\alpha =\;\pi /3,\;Ha=1,\;B_{1}=0.3,\;M_{1}=1.5$, $B_{2}=0.02,\;m_{e}=1,\;G_{t}=0.6,\;Sc=1.5,\;M_{2}=3,\;\gamma =0.6,\;Br=0.5$. (**e**): Effects of $B_{1}$ on u, while other parameters are $b_{1}=0.3,\;b_{2}=0.5,\;x=0.5,\;l=1,$ $\zeta_{1}=-0.5,\;\zeta_{2}=-0.4,\;U_{HS}=-0.75,\;\Theta =1.5,\;\Phi =\pi /3,\;\alpha =\;\pi /3,\;Ha=1,\;G_{t}=0.6,\;M_{1}=1,B_{2}=0.02,\;m_{e}=3,\;G_{a}=1.5,\;Sc=1.5,\;M_{2}=1,\;\gamma =0.6,\;Br=0.5$. (**f**): Effects of $B_{2}$ on u, while other parameters are $b_{1}=0.3,\;b_{2}=0.5,\;x=0.5,\;l=1,\;\zeta_{1}=-0.5,\;\zeta_{2}=-0.4,\;U_{HS}=-0.75,\;\Theta =1.5,\;\Phi =\pi /3,\;\alpha =\;\pi /3,\;Ha=1,\;B_{1}=0.2,\;M_{1}=1$, $G_{t}=0.6,\;m_{e}=1,\;G_{a}=0.8,\;Sc=1.5,\;M_{2}=1.5,\;\gamma =0.6,\;Br=0.5$.

**Figure 3:**
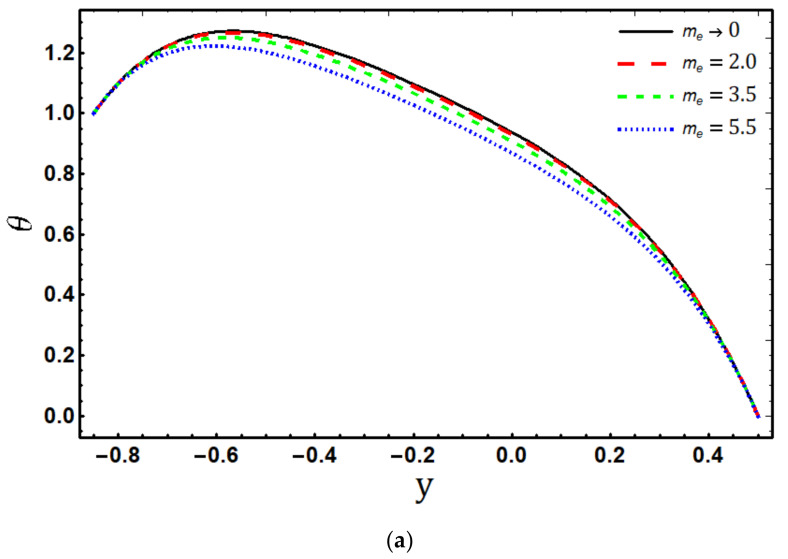

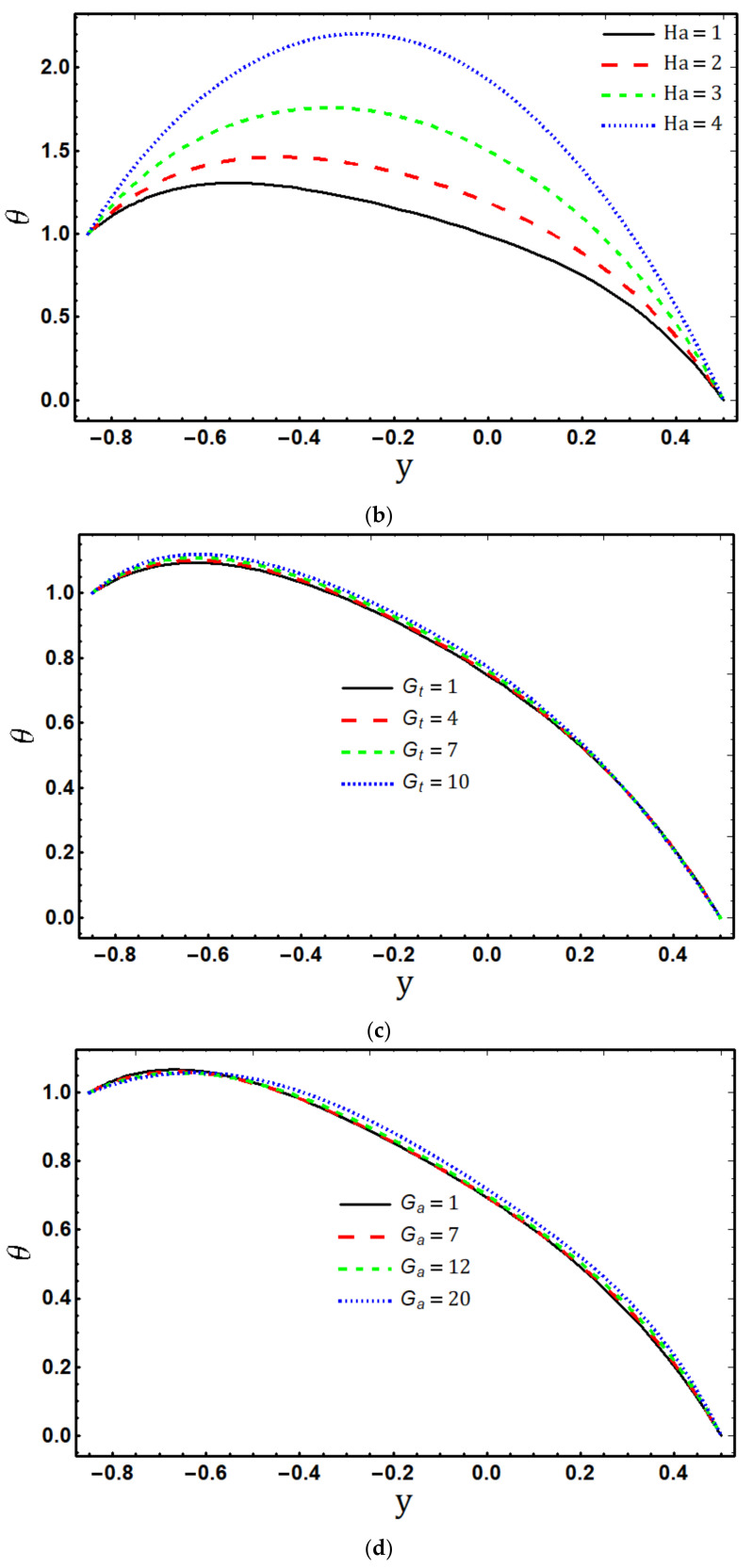

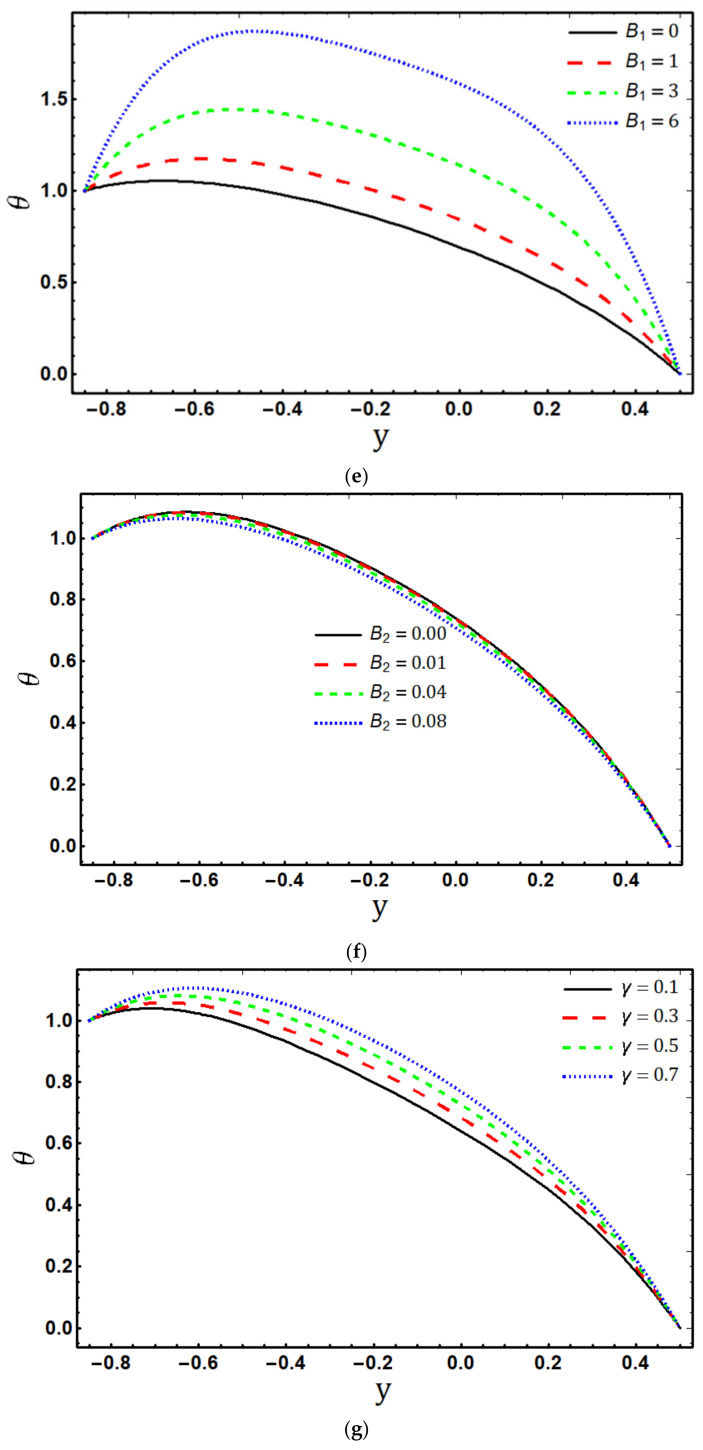

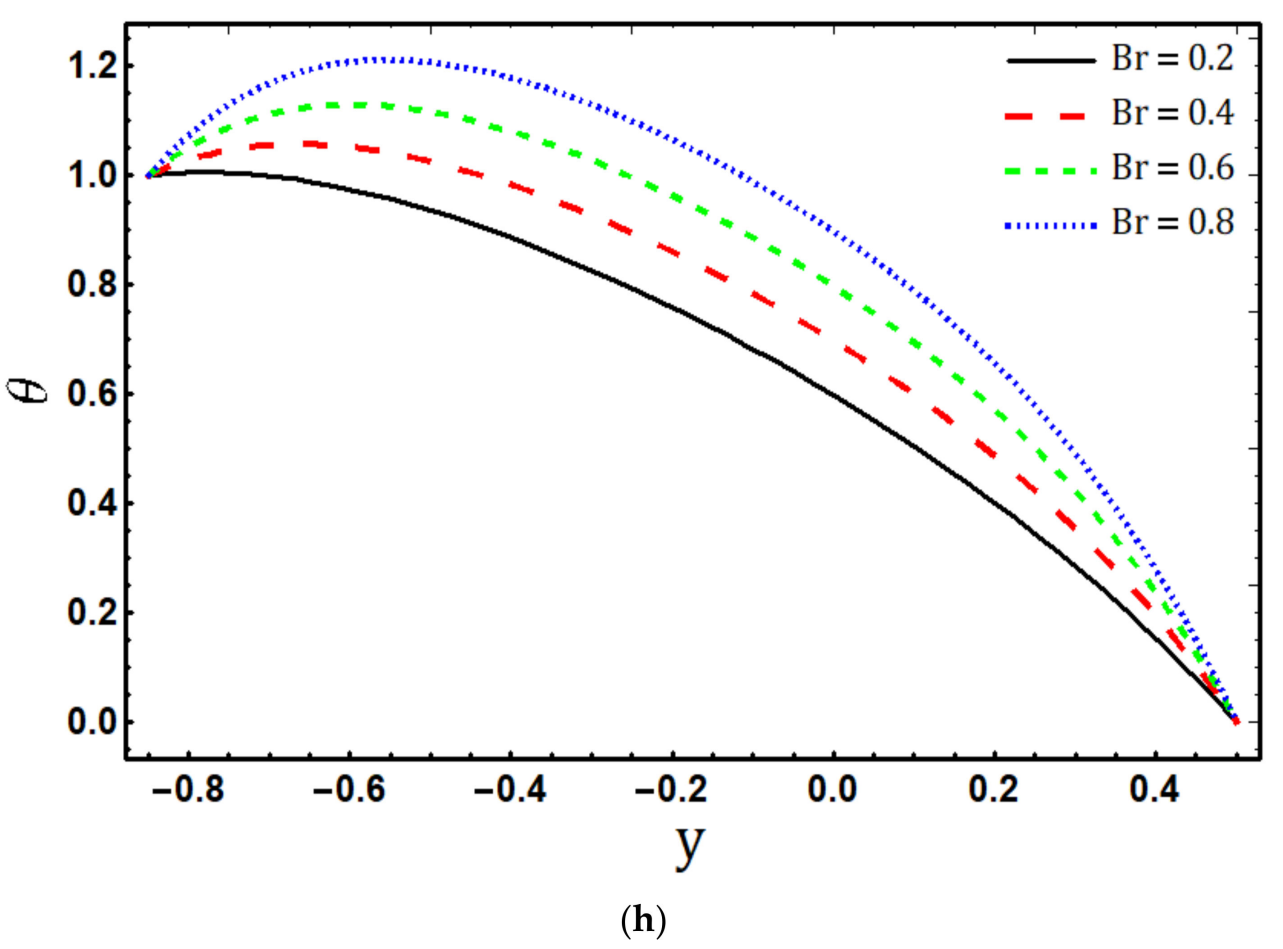
(**a**): Effects of $m_{e}$ on θ, while other parameters are $b_{1}=0.3,\;b_{2}=0.5,\;x=0.5,\;l=1,\zeta_{1}=-0.9,\;\zeta_{2}=-0.8,\;U_{HS}=-0.9,\;\Theta =1.5,\;\Phi =\pi /3,\;\alpha =\;\pi /3,\;Ha=0.1,\;B_{1}=2,\;M_{1}=1,B_{2}=0.02,\;G_{t}=0.6,\;G_{a}=0.5,\;Sc=1.5,\;M_{2}=2,\;\gamma =0.6,\;Br=0.5$. (**b**): Effects of Ha on θ, while other parameters are $b_{1}=0.3,\;b_{2}=0.5,\;x=0.5,\;l=1,\zeta_{1}=-0.5,\;\zeta_{2}=-0.4,\;U_{HS}=-0.75,\;\Theta =1.5,\;\Phi =\pi /3,\;\alpha =\;\pi /3,\;m_{e}=3,\;B_{1}=2,\;M_{1}=1$,$B_{2}=0.02,\;G_{t}=0.6,\;G_{a}=0.5,\;Sc=1.5,\;M_{2}=1.5,\;\gamma =0.6,\;Br=0.5$. (**c**): Effects of $G_{t}$ on θ, while other parameters are $b_{1}=0.3,\;b_{2}=0.5,\;x=0.5,\;l=1,\zeta_{1}=-0.5,\;\zeta_{2}=-0.4,\;U_{HS}=-0.5,\;\Theta =1.5,\;\Phi =\pi /3,\;\alpha =\;\pi /3,\;Ha=1,\;B_{1}=0.3,\;M_{1}=1.5,B_{2}=0.02,\;m_{e}=1,\;G_{a}=0.8,\;Sc=1.5,\;M_{2}=3,\;\gamma =0.6,\;Br=0.5$. (**d**): Effects of $G_{a}$ on θ, while other parameters are $b_{1}=0.3,\;b_{2}=0.5,\;x=0.5,\;l=1,\zeta_{1}=-0.5,\;\zeta_{2}=-0.4,\;U_{HS}=-0.5,\;\Theta =1.5,\;\Phi =\pi /3,\;\alpha =\;\pi /3,\;Ha=1,\;B_{1}=0.3,\;M_{1}=1.5$,$B_{2}=0.02,\;m_{e}=1,\;G_{t}=0.6,\;Sc=1.5,\;M_{2}=3,\;\gamma =0.6,\;Br=0.5$. (**e**): Effects of $B_{1}$ on θ, while other parameters are $b_{1}=0.3,\;b_{2}=0.5,\;x=0.5,\;l=1,\zeta_{1}=-0.5,\;\zeta_{2}=-0.4,\;U_{HS}=-0.75,\;\Theta =1.5,\;\Phi =\pi /3,\;\alpha =\;\pi /3,\;Ha=1,\;G_{t}=0.6,\;M_{1}=1,\;B_{2}=0.02,\;m_{e}=3,\;G_{a}=1.5,\;Sc=1.5,\;M_{2}=1,\;\gamma =0.6,\;Br=0.5$. (**f**): Effects of $B_{2}$ on θ, while other parameters are $b_{1}=0.3,\;b_{2}=0.5,\;x=0.5,\;l=1,\zeta_{1}=-0.5,\;\zeta_{2}=-0.4,\;U_{HS}=-0.75,\;\Theta =1.5,\;\Phi =\pi /3,\;\alpha =\;\pi /3,\;Ha=1,\;B_{1}=0.2,\;M_{1}=1$,$G_{t}=0.6,\;m_{e}=1,\;G_{a}=0.8,\;Sc=1.5,\;M_{2}=1.5,\;\gamma =0.6,\;Br=0.5$. (**g**): Effects of γ on θ, while other parameters are $b_{1}=0.3,\;b_{2}=0.5,\;x=0.5,\;l=1,\zeta_{1}=-0.5,\;\zeta_{2}=-0.4,\;U_{HS}=-0.5,\;\Theta =1.5,\;\Phi =\pi /3,\;\alpha =\;\pi /3,\;Ha=1,\;B_{1}=0.3,\;M_{1}=3$,$G_{t}=0.6,\;m_{e}=1,\;G_{a}=0.8,\;Sc=1.5,\;M_{2}=1.5,\;B_{2}=0.02,\;Br=0.5$. (**h**): Effects of Br on θ, while other parameters are $b_{1}=0.3,\;b_{2}=0.5,\;x=0.5,\;l=1,\zeta_{1}=-0.5,\;\zeta_{2}=-0.4,\;U_{HS}=-0.5,\;\Theta =1.5,\;\Phi =\pi /3,\;\alpha =\;\pi /3,\;Ha=1,\;B_{1}=0.3,\;M_{1}=3,G_{t}=0.6,\;m_{e}=1,\;G_{a}=0.8,\;Sc=1.5,\;M_{2}=1.5,\;\gamma =0.6,\;B_{2}=0.02$.

**Figure 4:**
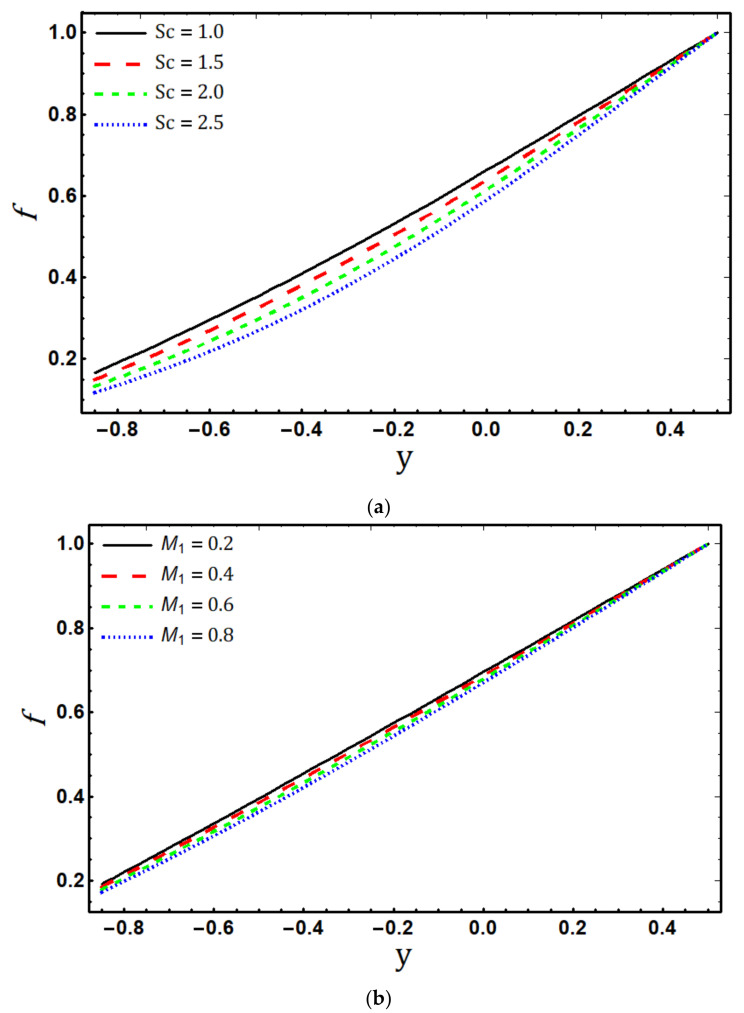

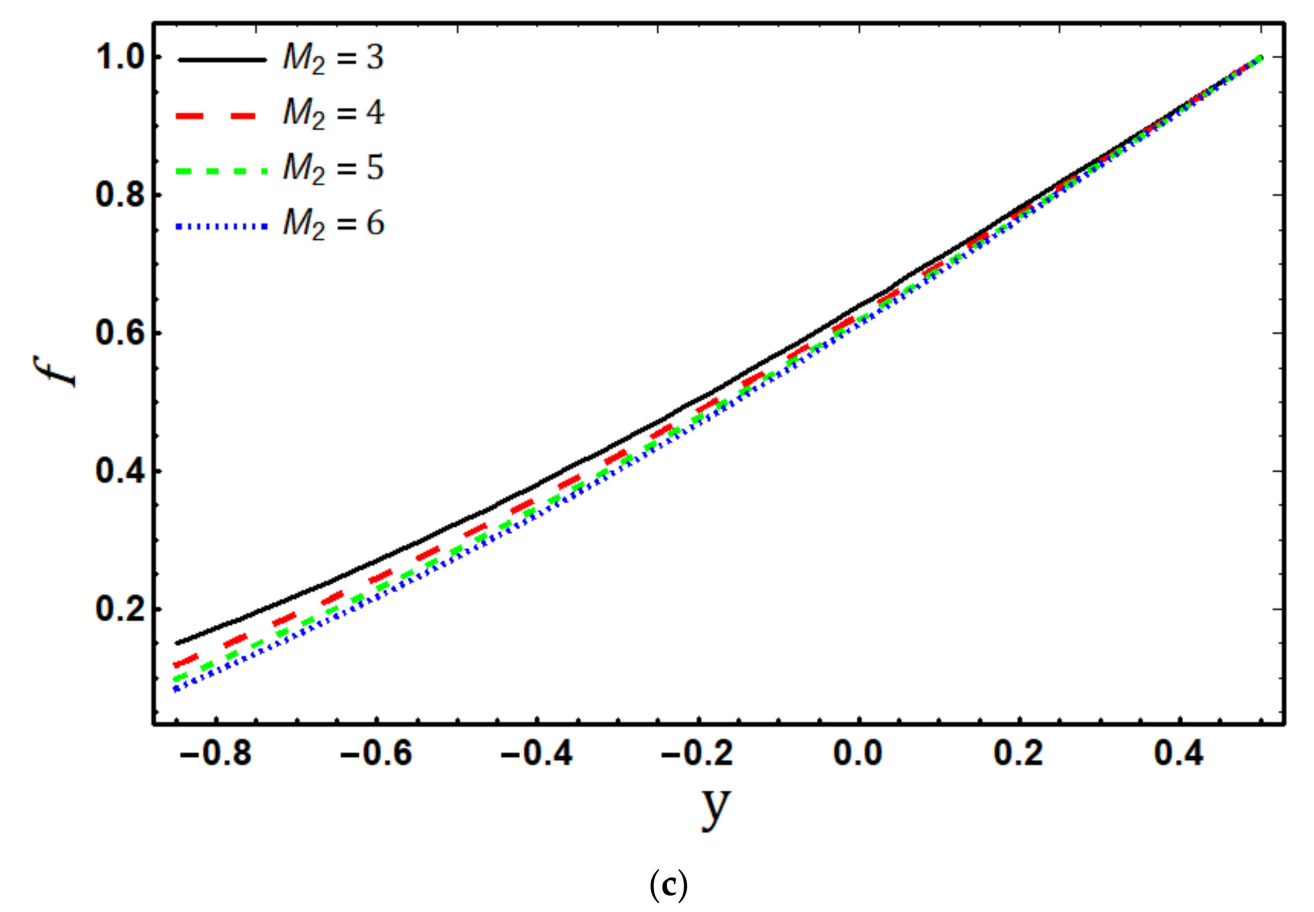
(**a**): Effects of Sc on f, while other parameters are $b_{1}=0.3,\;b_{2}=0.5,\;x=0.5,\;l=1,\zeta_{1}=-0.5,\;\zeta_{2}=-0.4,\;U_{HS}=-0.5,\;\Theta =1.5,\;\Phi =\pi /3,\;\alpha =\;\pi /3,\;Ha=1,\;B_{1}=0.3,\;M_{1}=3,G_{t}=0.6,\;m_{e}=1,\;G_{a}=0.8,\;B_{2}=0.02,\;M_{2}=1.5,\;\gamma =0.6,\;Br=0.5$. (**b**): Effects of $M_{1}$ on f, while other parameters are $b_{1}=0.3,\;b_{2}=0.5,\;x=0.5,\;l=1,\zeta_{1}=-0.5,\;\zeta_{2}=-0.4,\;U_{HS}=-0.5,\;\Theta =1.5,\;\Phi =\pi /3,\;\alpha =\;\pi /3,\;Ha=1,\;B_{1}=0.3,\;B_{2}=0.02$,$G_{t}=0.6,\;m_{e}=1,\;G_{a}=0.8,\;Sc=1.5,\;M_{2}=1.5,\;\gamma =0.6,\;Br=0.5$. (**c**): Effects of $M_{2}$ on f, while other parameters are $b_{1}=0.3,\;b_{2}=0.5,\;x=0.5,\;l=1,\zeta_{1}=-0.5,\;\zeta_{2}=-0.4,\;U_{HS}=-0.5,\;\Theta =1.5,\;\Phi =\pi /3,\;\alpha =\;\pi /3,\;Ha=1,\;B_{1}=0.2,\;M_{1}=0.6,G_{t}=0.6,\;m_{e}=1,\;G_{a}=0.8,\;Sc=1.5,\;B_{2}=0.02,\;\gamma =0.6,\;Br=0.5$.

**Figure 5:**
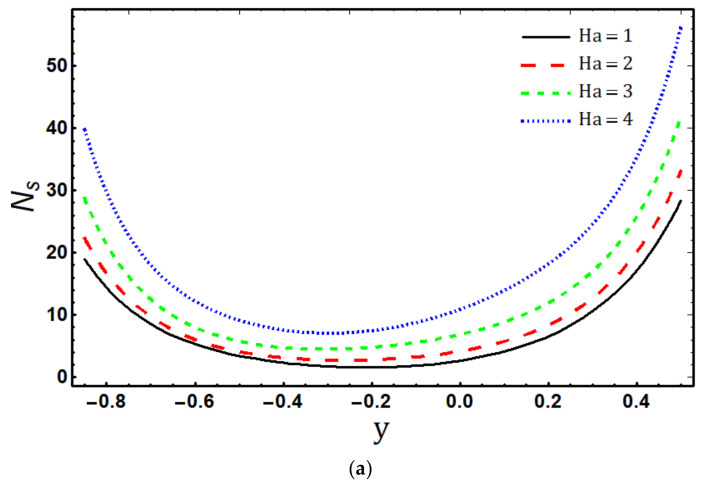

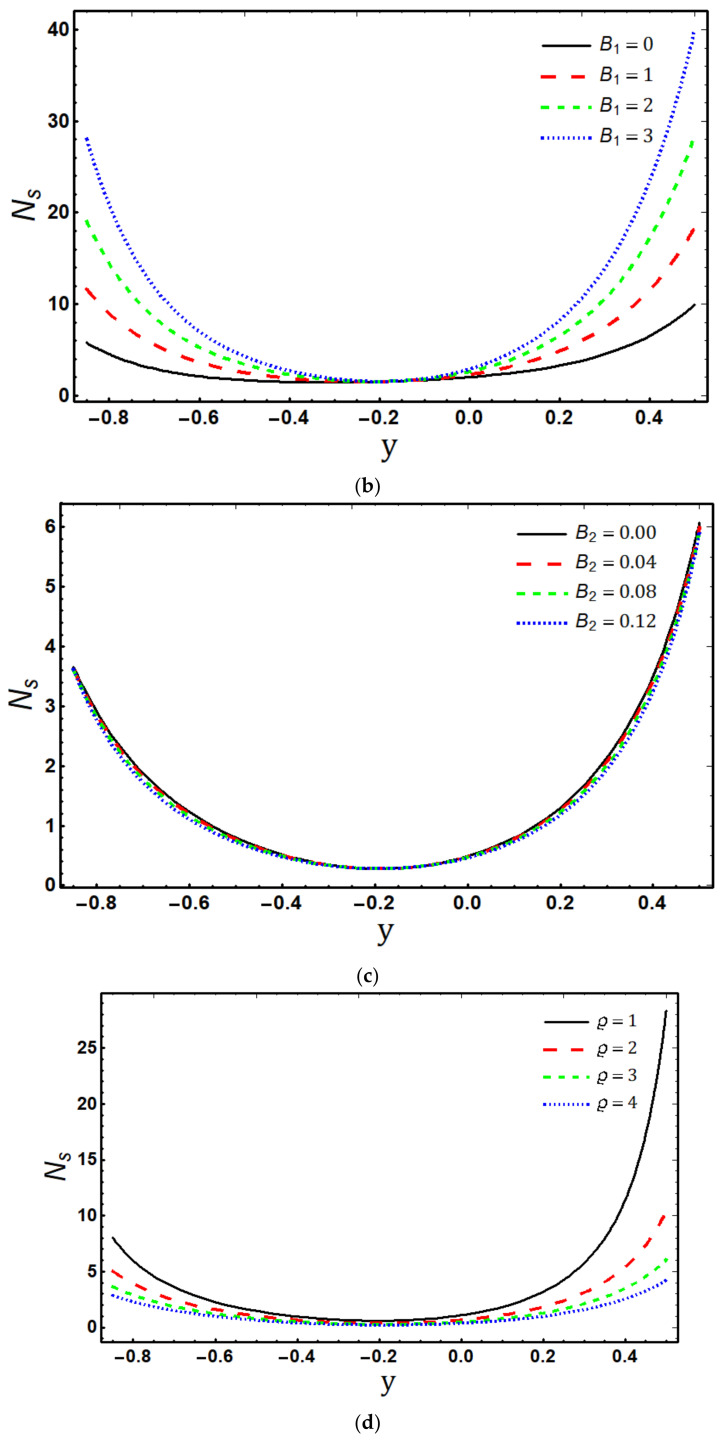

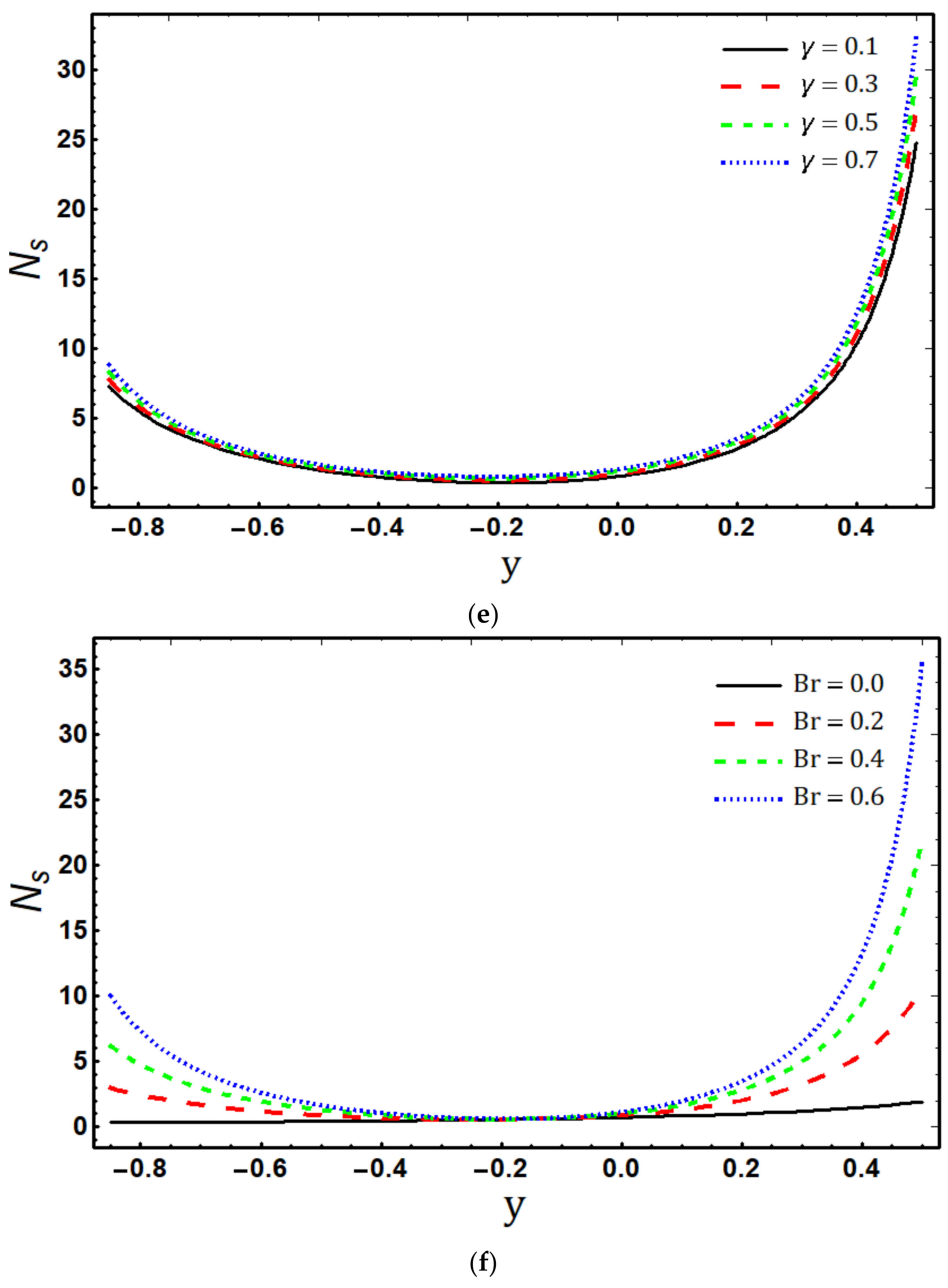
(**a**): Effects of Ha on $N_{s}$, while other parameters are $b_{1}=0.3,\;b_{2}=0.5,\;x=0.5,\;l=1,\zeta_{1}=-0.5,\;\zeta_{2}=-0.4,\;U_{HS}=-0.75,\;\Theta =1.5,\;\Phi =\pi /3,\;\alpha =\;\pi /3,\;m_{e}=3,\;B_{1}=2,\;M_{1}=1,B_{2}=0.02,\;G_{t}=0.6,\;G_{a}=1.5,\;Sc=1.5,\;M_{2}=1.5,\;\gamma =0.6,\;Br=0.5,\;\varrho =1$. (**b**): Effects of $B_{1}$ on $N_{s}$, while other parameters are $b_{1}=0.3,\;b_{2}=0.5,\;x=0.5,\;l=1,\zeta_{1}=-0.5,\;\zeta_{2}=-0.4,\;U_{HS}=-0.75,\;\Theta =1.5,\;\Phi =\pi /3,\;\alpha =\;\pi /3,\;Ha=1,\;G_{t}=0.6,\;M_{1}=1,B_{2}=0.02,\;m_{e}=3,\;G_{a}=1.5,\;Sc=1.5,\;M_{2}=1.5,\;\gamma =0.6,\;Br=0.5,\;\varrho =1$. (**c**): Effects of $B_{2}$ on $N_{s}$, while other parameters are $b_{1}=0.3,\;b_{2}=0.5,\;x=0.5,\;l=1,\zeta_{1}=-0.5,\;\zeta_{2}=-0.4,\;U_{HS}=-0.75,\;\Theta =1.5,\;\Phi =\pi /3,\;\alpha =\;\pi /3,\;Ha=1,\;B_{1}=2,\;M_{1}=1$,$G_{t}=0.6,\;m_{e}=1,\;G_{a}=0.8,\;Sc=1.5,\;M_{2}=1.5,\;\gamma =0.6,\;Br=0.5,\;\varrho =3$. (**d**): Effects of ϱ on $N_{s}$, while other parameters are $b_{1}=0.3,\;b_{2}=0.5,\;x=0.5,\;l=1,\zeta_{1}=-0.5,\;\zeta_{2}=-0.4,\;U_{HS}=-0.75,\;\Theta =1.5,\;\Phi =\pi /3,\;\alpha =\;\pi /3,\;Ha=1,\;B_{1}=2,\;M_{1}=1$,$G_{t}=0.6,\;m_{e}=3,\;G_{a}=0.8,\;Sc=1.5,\;M_{2}=1.5,\;\gamma =0.6,\;Br=0.5,\;B_{2}=0.02$. (**e**): Effects of γ on $N_{s}$, while other parameters are $b_{1}=0.3,\;b_{2}=0.5,\;x=0.5,\;l=1,\zeta_{1}=-0.5,\;\zeta_{2}=-0.4,\;U_{HS}=-0.75,\;\Theta =1.5,\;\Phi =\pi /3,\;\alpha =\;\pi /3,\;Ha=1,\;B_{1}=0.2,\;M_{1}=1$,$G_{t}=0.6,\;m_{e}=3,\;G_{a}=1.5,\;Sc=1.5,\;M_{2}=1.5,\;B_{2}=0.02,\;Br=0.5,\;\varrho =1$. (**f**): Effects of Br on $N_{s}$, while other parameters are $b_{1}=0.3,\;b_{2}=0.5,\;x=0.5,\;l=1,\zeta_{1}=-0.5,\;\zeta_{2}=-0.4,\;U_{HS}=-0.5,\;\Theta =1.5,\;\Phi =\pi /3,\;\alpha =\;\pi /3,\;Ha=1,\;B_{1}=2,\;M_{1}=1,G_{t}=0.6,\;m_{e}=3,\;G_{a}=1.5,\;Sc=1.5,\;M_{2}=1.5,\;\gamma =0.6,\;B_{2}=0.02,\;\varrho =1$.

**Figure 6:**
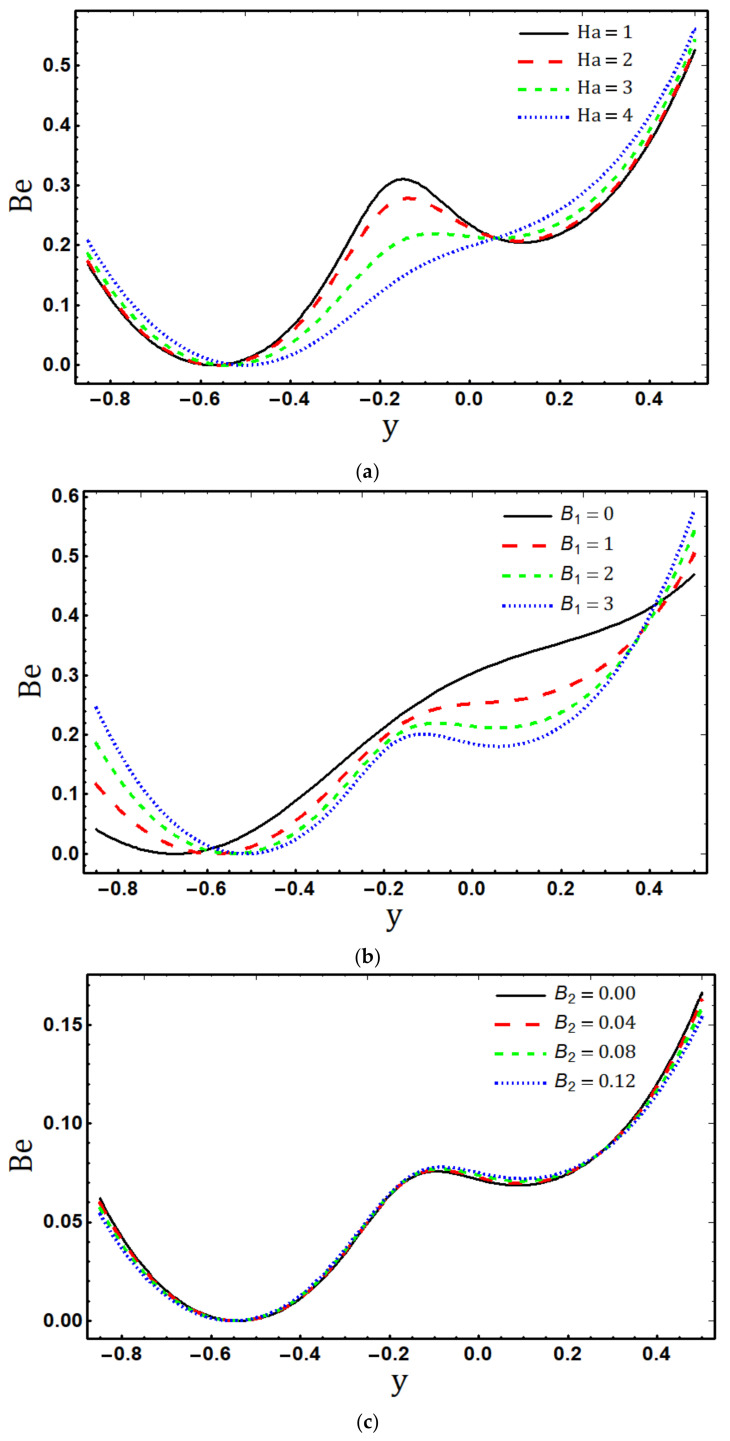

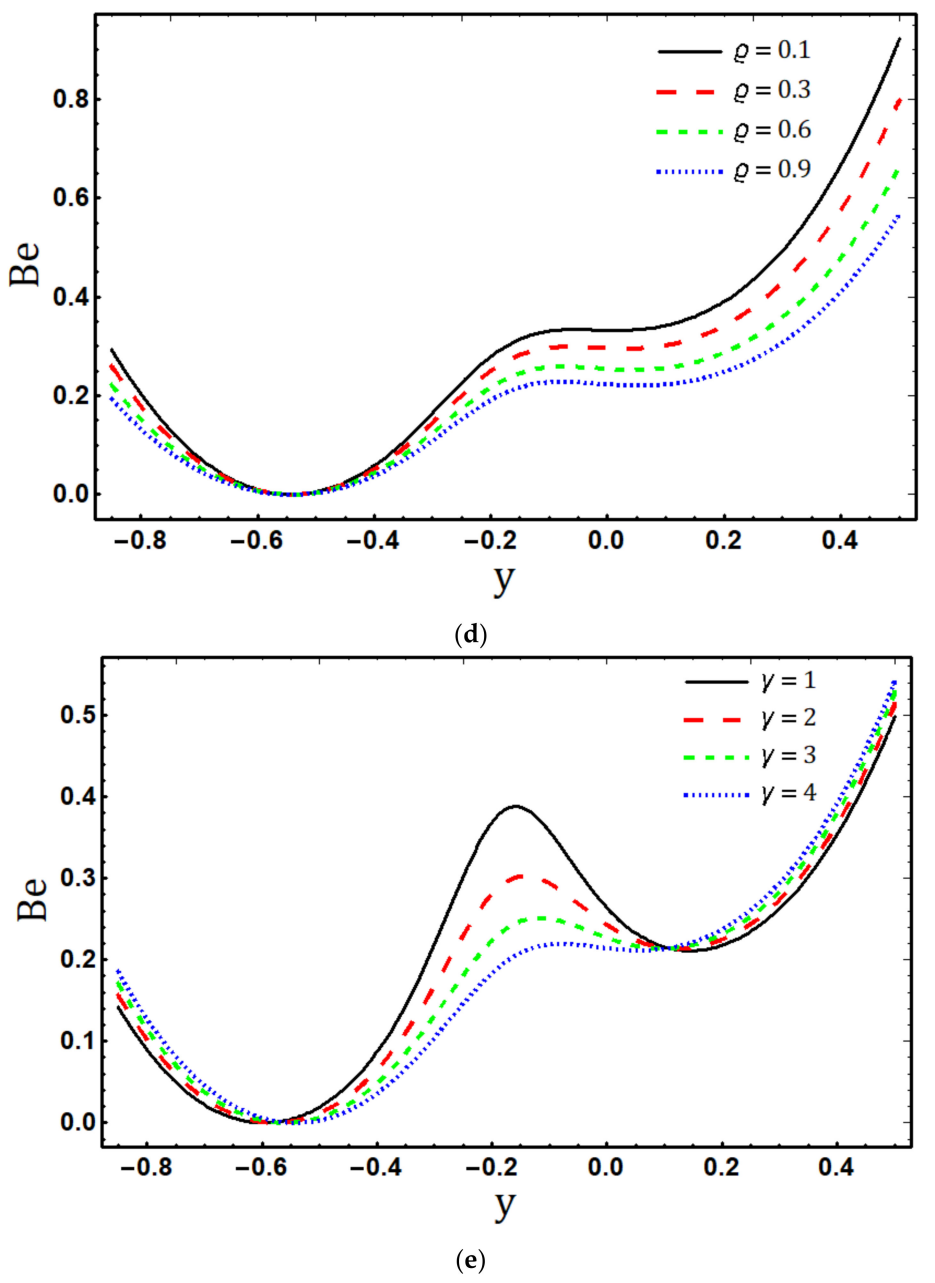

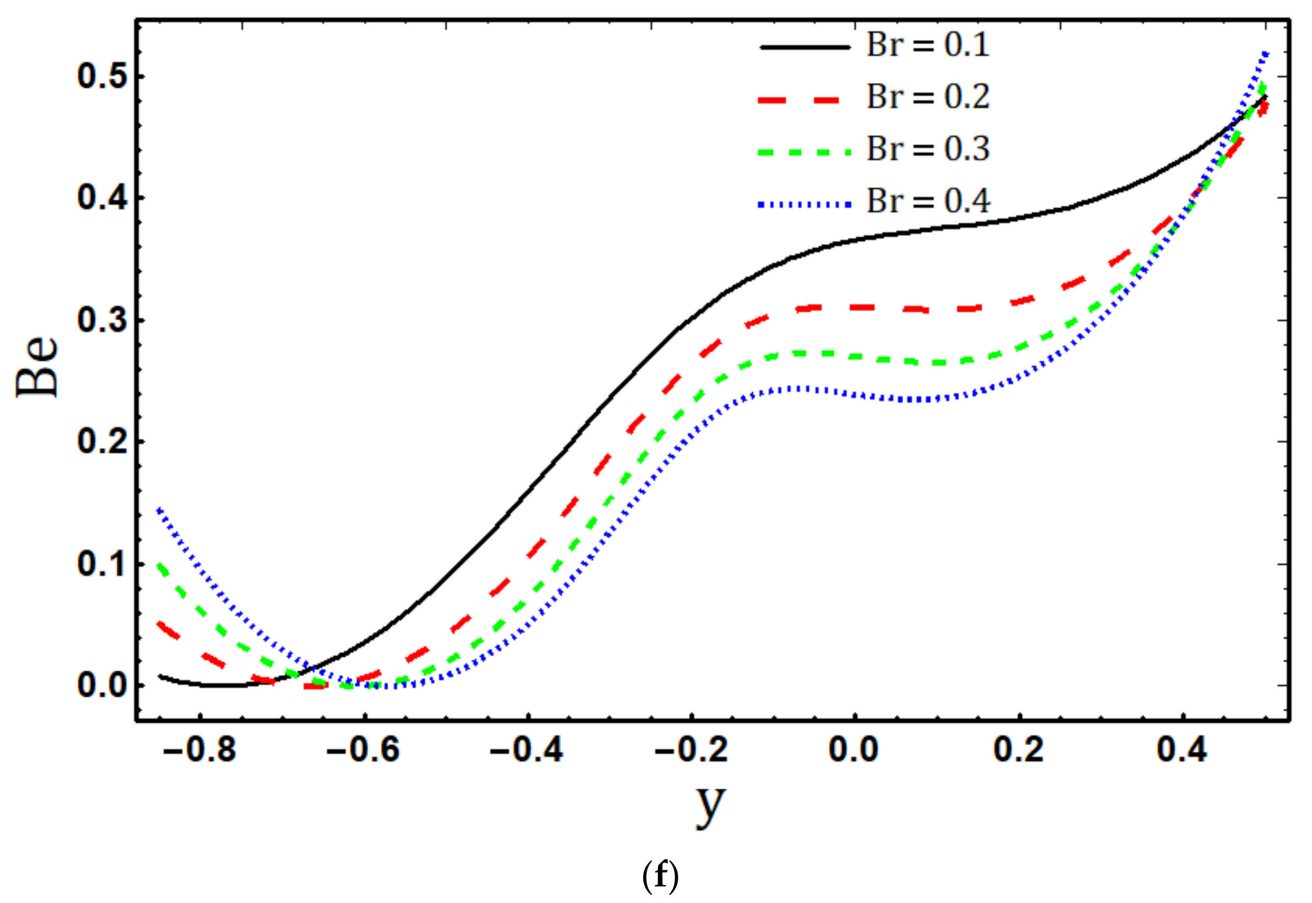
(**a**): Effects of Ha on Be, while other parameters are $b_{1}=0.3,\;b_{2}=0.5,\;x=0.5,\;l=1,\zeta_{1}=-0.5,\;\zeta_{2}=-0.4,\;U_{HS}=-0.75,\;\Theta =1.5,\;\Phi =\pi /3,\;\alpha =\;\pi /3,\;m_{e}=3,\;B_{1}=2,\;M_{1}=1$,$B_{2}=0.02,\;G_{t}=0.6,\;G_{a}=1.5,\;Sc=1.5,\;M_{2}=1.5,\;\gamma =0.6,\;Br=0.5,\;\varrho =1$. (**b**): Effects of $B_{1}$ on Be, while other parameters are $b_{1}=0.3,\;b_{2}=0.5,\;x=0.5,\;l=1,\zeta_{1}=-0.5,\;\zeta_{2}=-0.4,\;U_{HS}=-0.75,\;\Theta =1.5,\;\Phi =\pi /3,\;\alpha =\;\pi /3,\;Ha=1,\;G_{t}=0.6,\;M_{1}=1,B_{2}=0.12,\;m_{e}=3,\;G_{a}=1.5,\;Sc=1.5,\;M_{2}=1.5,\;\gamma =0.6,\;Br=0.5,\;\varrho =1$. (**c**): Effects of $B_{2}$ on Be, while other parameters are $b_{1}=0.3,\;b_{2}=0.5,\;x=0.5,\;l=1,\zeta_{1}=-0.5,\;\zeta_{2}=-0.4,\;U_{HS}=-0.75,\;\Theta =1.5,\;\Phi =\pi /3,\;\alpha =\;\pi /3,\;Ha=1,\;B_{1}=2,\;M_{1}=1$,$G_{t}=0.6,\;m_{e}=1,\;G_{a}=1.5,\;Sc=1.5,\;M_{2}=1.5,\;\gamma =0.6,\;Br=0.5,\;\varrho =6$. (**d**): Effects of ϱ on Be, while other parameters are $b_{1}=0.3,\;b_{2}=0.5,\;x=0.5,\;l=1,\zeta_{1}=-0.5,\;\zeta_{2}=-0.4,\;U_{HS}=-0.75,\;\Theta =1.5,\;\Phi =\pi /3,\;\alpha =\;\pi /3,\;Ha=1,\;B_{1}=2,\;M_{1}=1,G_{t}=0.6,\;m_{e}=3,\;G_{a}=0.8,\;Sc=1.5,\;M_{2}=1.5,\;\gamma =0.6,\;Br=0.5,\;B_{2}=0.02$. (**e**): Effects of γ on Be, while other parameters are $b_{1}=0.3,\;b_{2}=0.5,\;x=0.5,\;l=1,\zeta_{1}=-0.5,\;\zeta_{2}=-0.4,\;U_{HS}=-0.75,\;\Theta =1.5,\;\Phi =\pi /3,\;\alpha =\;\pi /3,\;Ha=1,\;B_{1}=0.2,\;M_{1}=1$,$G_{t}=0.6,\;m_{e}=3,\;G_{a}=1.5,\;Sc=1.5,\;M_{2}=1.5,\;B_{2}=0.02,\;Br=0.5,\;\varrho =1$. (**f**): Effects of Br on Be, while other parameters are $b_{1}=0.3,\;b_{2}=0.5,\;x=0.5,\;l=1,\zeta_{1}=-0.5,\;\zeta_{2}=-0.4,\;U_{HS}=-0.75,\;\Theta =1.5,\;\Phi =\pi /3,\;\alpha =\;\pi /3,\;Ha=1,\;B_{1}=2,\;M_{1}=1,G_{t}=0.6,\;m_{e}=3,\;G_{a}=1.5,\;Sc=1.5,\;M_{2}=1.5,\;\gamma =0.6,\;B_{2}=0.02,\;\varrho =1$.

In this section, the influence of several physical parameters on entropy generation number, Bejan number, temperature, velocity, and contribution distribution of an Eyring–Powell fluid via inclined asymmetric channel are presented graphically through a set of Figure 2, Figure 3, Figure 4, Figure 5 and Figure 6.

### 5.1. Velocity Profile

Figure 2a–f shows the effects of the electroosmotic parameter $m_{e},$ Hartmann number Ha, Eyring–Powell fluid parameters $B_{1}$ and $B_{2}$, temperature Grashoff number $G_{t}$ and mass Grashoff number $G_{a}$ on the velocity profile u as a function of transverse coordinate y. Figure 2a demonstrates that the Eyring–Powell fluid axial velocity u decelerates in the channel’s centre and accelerates near the channel walls as the Debye–Huckel parameter $m_{e}$ increases. As the electroosmotic parameter $m_{e}$ rises, the EDL thickness decreases, and it is inferred that the electric body force will be considerably greater towards the channel’s boundary than in its centre, causing the velocity to increase in the region near the channel walls. Additionally, because the lower wall $\left(\zeta_{1}=-0.9\right)$ has a smaller zeta potential than the upper wall $\left(\zeta_{2}=-0.8\right)$, the velocity is higher near the lower wall. The behavior of the velocity field u under the influence of an external magnetic field is depicted in Figure 2b. By raising the Hartmann number Ha, it is shown that fluid flow is hindered in the centre and boosted at the walls. This fact is caused by a magnetic field’s anisotropic properties, which resist motion that is normal to it while permitting motion parallel to it to continue unimpeded. Figure 2c shows that for different values of the temperature Grashoff number $G_{t}$, the behavior of the velocity distribution u is not symmetric. The Eyring–Powell fluid velocity u is observed to increase near the lower wall and decrease near the upper wall when $G_{t}$ enhances. It is due to the fact that the lower section of the fluid has less fluid viscosity because of the greater temperature at the lower wall $\left(T_{1}>T_{0}\right)$, which accelerates velocity. By rising the mass Grashof number $G_{a}$, it can be seen in Figure 2d that the velocity increases at the upper section and drops near the bottom portion of the asymmetric channel. This observation is comparable to the findings of the study [16]. According to Figure 2e, the axial velocity u in the middle of the microchannel increases as the material fluid parameter $B_{1}$ increases, but it decreases close to the channel walls. This is because that the Eyring–Powell fluid becomes thin in the centre due to decreased viscosity, whereas viscous effects are more pronounced close to the walls. Figure 2f indicates that the behavior of Eyring–Powell fluid parameter $B_{2}$ on the velocity distribution is opposite to $B_{1}$. Physically, the Eyring–Powell fluid’s thickness is enhanced by increasing its dynamic viscosity and the velocity u decelerated. It is important to note that the Newtonian fluid is represented by the curve $B_{1}=B_{2}=0$ in Figure 2e,f.

### 5.2. Temperature Profile

Figure 3a–h shows the effects of emerging parameters on temperature profile θ for flow in a microchannel with walls set at different constant temperatures. It is obvious that the temperature θ curves decrease as the transverse coordinate y increases in order to satisfy the boundary conditions. Figure 3a gives the effect of the EDL thickness on the temperature profile θ along the height of the channel. The fluid’s temperature θ drops due to the electroosmotic parameter $m_{e}$. The magnetic field causes the temperature θ of the Eyring–Powell fluid to rise throughout the channel, depicted in Figure 3b. For larger Hartmann number Ha, the Lorentz force boosts up, which causes more resistance to the flow. Therefore, temperature θ rises as a result of the conversion of fluid particle kinetic energy into heat energy. The fluid temperature θ rises as a result of the impacts of both heat Grashof number $G_{t}$ and mass Grashof numbers $G_{a}$, as seen in Figure 3c,d. This is due to the decline in viscous forces. As the relationship between temperature and viscosity is inverse, a rise in temperature θ is caused by a reduction in viscous forces. The descriptions of the Eyring–Powell fluid parameters $B_{1}$ and $B_{2}$ on the temperature distribution θ are shown in Figure 3e,f. It is observed that enhancing $B_{1}$ rises fluid temperature, whereas increasing $B_{2}$ lessens fluid temperature. Physically, the increased value of $B_{1}$ decreases the dynamic viscosity, which causes the temperature to rise, and the increment of $B_{2}$ improves the dynamic viscosity, which causes the temperature to fall. According to Figure 3g,h, the temperature field θ is an increasing function for both viscous dissipation paramater Br and Ohmic dissipation parameter γ. Since both the Brinkman number Br (which produces heat via shear stress) and the Joule heating parameter γ (which produces heat through electric and magnetic fields) are related to the creation of heat, as Br and γ are boosted, more heat is generated, which causes the Eyring–Powell fluid’s temperature θ to rise.

### 5.3. Concentration Profile

Figure 4a–c gives the graphical results for the variation of concentration distribution f with different values of Schmidt number Sc, homogeneous reaction parameter $M_{1}$, and heterogeneous reaction parameter $M_{2}$. The concentration distribution f for the Schmidt number Sc, homogeneous reaction parameter $M_{1}$, and heterogeneous reaction parameter $M_{2}$ are shown in Figure 4a–c. Figure 4a reveals that concentration field f is a decreasing function of Schmidt parameter Sc. Physically, when the Schmidt number Sc rises, the density of the Eyring–Powell fluid particle diminishes. So, the less dense fluid particles are moving at a faster rate and distributing more molecules; consequently, the concentration f drops. Figure 4b elucidates that the concentration f diminished with the effects of the homogeneous reaction parameter $M_{1}$. Since the solute reaction rate is sped up by increasing the homogeneous reaction parameter $M_{1}$ as a result of a fall in fluid viscosity, which decreases the reactant concentration, the reduction in concentration f is seen in Figure 4c for a greater heterogeneous reaction parameter $M_{2}$. Since the heterogeneous reaction parameter $M_{2}$ has a direct proportionality to the reaction rate, the concentration of species decreases as $M_{2}$ rises as a consequence of an increase in reaction rate and a reduction in diffusion rate.

### 5.4. Entropy Generation

Figure 5a–f is plotted to illustrate the impact of Ha, $B_{1}$, $B_{2}$ ϱ, γ, and Br on total entropy generation $N_{s}$ because an irreversibility analysis is required to determine the system’s efficiency. These figures show that the entropy generation profiles $N_{s}$ are parabolic, with higher values at the ends of channel walls and lower values in the centre. Furthermore, the entropy generation number $N_{s}$ is lower in the lower portion of the channel than in the upper portion of the channel due to low zeta potential values at the lower wall and the nature of the asymmetric channel. The entropy generation number $N_{s}$ increases as the magnetic field strength increases, as seen in Figure 5a. The reason for this is that the thermal irreversibility $N_{s}$ and the Hartmann number Ha are directly related. Figure 5b explored that the entropy generation number $N_{s}$ grows quickly close to the channel walls while remaining constant near the centre for larger values of material fluid parameter $B_{1}$ since fluid temperature θ rises with increase in $B_{1}$ and entropy is directly related to temperature also increases. With an increase in the material fluid parameter $B_{2}$, Figure 5c reveals that the total entropy generation $N_{s}$ decreases. This is related to the damping impact of $B_{2}$ on fluid flow, which reduces entropy in the microchannel. Figure 5d displays the change in thermal irreversibility for various values of temperature difference parameter ϱ. It has been shown that $N_{s}$ declines as ϱ increases. According to Figure 5e,f, when the Ohmic dissipation parameter γ and viscous dissipation parameter Br increase, the total entropy generation $N_{s}$ rises along the height of the channel. Increases in the Joule heating parameter γ and Brinkman number Br imply an increase in heat generation through dissipation, which raises irreversibility within the channel. The fluctuation of the Bejan number caused by $Ha,\;B_{1},\;B_{2},\;\varrho ,\;\gamma$, and Br is examined in Figure 6a–f. These findings reveal that Be=0, near the bottom wall, which denotes irreversibility caused by the combined effects of viscous dissipation, electric field, and magnetic field, is prevalent. In Figure 6a, it is obvious that as the Hartmann number Ha rises, the Bejan number Be diminishes in the centre and increases close to the channel’s boundaries because the irreversibility caused by the magnetic field in the channel’s core is more immense than the irreversibility caused by heat transfer. Figure 6b expresses that the magnitude of Be profile increases near the lower and upper walls of the channel, while the opposite behavior is observed in the centre region. When $B_{1}$ increases, the fluid becomes thick and irreversibly due to viscosity of fluid near the walls is higher than the heat transfer irreversibility. From the Figure 6c, the magnitude of the Be profile diminishes at the bottom and top borders of the channel, whereas the opposite behavior is seen in the centre. This is because irreversibility caused by fluid viscosity close to walls is greater than irreversibility caused by heat transfer. It is observed from Figure 6d that Be decreases as the Temperature difference parameter ϱ increases. The fluctuation in heat transfer irreversibility in relation to the total irreversibility caused by  γ and Br is shown in Figure 6e–f. The rate of entropy formation is larger in the centre—due to Ohmic, magnetic, and viscous dissipation—than it is near to the walls, where heat transfer irreversibility predominates due to a significant temperature gradient.

In Table 1, the entropy generation numbers $N_{s}$ for Eyring–Powell fluid and viscous fluid are contrasted for three different values of Ha, Br, γ, and ϱ. Due to the decreased viscous forces, Eyring–Powell fluid (shear thinning fluid) has less entropy than viscous fluid.

## 6. Concluding Remarks

The primary focus of this work has been on the entropy generation analysis for the electrokinetic flow of Eyring–Powell fluid in an inclined asymmetric microchannel. Study is performed under the physical effects of the magnetic field, viscous dissipation, electric field, mixed convection, Joule heating, and homogeneous and heterogeneous reactions. Drug delivery, DNA sequencing, and medical diagnosis are just a few fields that could benefit from electro-osmotic peristaltic flows. These systems can be designed and optimized with the help of entropy generation analysis. The governing equations are modified with the help of reliable approximation. The solutions to the governing flow equations are numerically estimated for flow, concentration, and temperature. Graphs are used to examine concentration, velocity, and temperature fields as well as Bejan and entropy generation numbers for various relevant non-dimensional parameters such as electroosmotic parameter $m_{e},$ Hartmann number Ha, Eyring–Powell fluid parameters $B_{1}$ and $B_{2}$, temperature Grashoff number $G_{t}$, mass Grashoff number $G_{a}$, viscous dissipation paramater Br, Ohmic dissipation parameter γ, Schmidt number Sc, homogeneous reaction parameter $M_{1}$, and heterogeneous reaction parameter $M_{2}$, accompanied by a detailed scientific explanation. Further, the numerical outcomes of the entropy generation of the Eyring–Powell fluid and the Newtonian fluid are examined in Table 1. Significant observations are summarized below:

- The axial velocity distribution u displays divergent behaviour in the centre of the channel and near to its boundaries as the values of the material fluid parameters $B_{1}$ and $B_{2}$ increase;
- The fluid temperature θ increases by increasing $Ha,\;B_{1},\;G_{t}$, and $G_{a}$, while it decreases for $m_{e}$ and $B_{2}$;
- Temperature θ is the increasing function of viscous dissipation parameter Br and Joule heating parameter γ;
- The concentration field f declines as the Schmidt number Sc rises;
- Heterogeneous reaction parameter $M_{2}$ enhances the concentration field f while the homogenous reaction parameter $M_{1}$ reduces the concentration f;
- For $B_{1}=0$ and $B_{2}=0$, the current research may be condensed to viscous fluid flow.
- The entropy generation rate increases with increasing $Br,\;\gamma ,\;B_{1}$, and Ha and decreases with increasing $B_{2}$ and ϱ;
- Maximum entropy production $N_{s}$ occurs in the vicinity of the channel walls;
- The dominance of the electric field, fluid friction, and magnetic field irreversibility is represented by Be=0 near the lower wall in the Bejan number profile.

## Figures and Tables

**Table 1:** Comparison for entropy generation rate $N_{s}$ between Eyring–Powell fluid and viscous fluid when and other fixed parameters are $b_{1}=0.3,\;b_{2}=0.5,\;x=0.5,\;l=1,\;U_{HS}=-0.75,\zeta_{1}=-0.5,\;\zeta_{2}=-0.4,\;U_{HS}=-0.75,\;\Theta =1.5,\;\Phi =\pi /3,\;\alpha =\pi /3,\;m_{e}=3,M_{1}=1,M_{2}=1.5,G_{t}=0.6,\;G_{a}=1.5,\;Sc=1.5$.

Ha	Br	γ	ϱ	Eyring–Powell Fluid $\left(N_{s}\right)$	Viscous Fluid $\left(N_{s}\right)$
1	0.4	0.6	1	0.619415	0.677288
2	0.4	0.6	1	1.06133	1.14184
3	0.4	0.6	1	1.64373	1.71052
1	0.0	0.6	1	0.524283	0.524283
1	0.2	0.6	1	0.565297	0.58877
1	0.4	0.6	1	0.602348	0.648808
1	0.4	0.0	1	0.388873	0.418463
1	0.4	0.4	1	0.545421	0.59462
1	0.4	0.8	1	0.690755	0.756632
1	0.4	0.6	1	0.619415	0.677288
1	0.4	0.6	2	0.405706	0.416037
1	0.4	0.6	3	0.301105	0.299335

## Data Availability

All data related to this research is available in manuscript.

## Nomenclature

**Symbol**	**Meaning**	**Dimensions**
${\bar{H}}_{1}\left(\bar{X},\bar{t}\right),\;{\bar{H}}_{2}\left(\bar{X},\;\bar{t}\right)$	Dimensional lower and upper walls in the fixed frame	$\left[L\right]$
${\bar{h}}_{1}\left(\bar{x}\right),\;{\bar{h}}_{2}\left(\bar{x}\right)$	Dimensional lower and upper walls in the wave frame	$\left[L\right]$
${\bar{b}}_{1},\;{\bar{b}}_{2}$	Dimensional amplitude of the wavy walls in the fixed frame	$\left[L\right]$
$b_{1},\;b_{2}$	Dimensionless amplitude of the wavy walls in the wave frame	$\left[-\right]$
c	Speed of wave	$\left[LT^{-1}\right]$
$l_{1}+l_{2}$	Width of the channel	$\left[L\right]$
$\bar{t}$	Dimensional time in fixed frame	$\left[T\right]$
λ	Wavelength	$\left[L\right]$
$\bar{X},\bar{Y}$	Dimensional coordinates in the fixed frame	$\left[L\right]$
$\bar{x},\bar{y}$	Dimensional coordinates in the wave frame	$\left[L\right]$
x,y	Dimensionless coordinates	$\left[-\right]$
$\bar{U},\bar{V}$	Dimensional velocity components in the fixed frame	$\left[LT^{-1}\right]$
$\bar{u},\bar{v}$	Dimensional velocity components in the wave frame	$\left[LT^{-1}\right]$
u,v	Dimensionless velocity components	$\left[-\right]$
$\alpha_{0}$	Angle of inclination	$\left[-\right]$
Φ	Phase difference	$\left[-\right]$
$T_{0},T_{1}$	Dimensional upper and lower wall temperature	$\left[K\right]$
θ	Dimensionless temperature $\left[-\right]$	$\left[-\right]$
A,B	Chemical species	$\left[-\right]$
$\bar{a},\bar{b}$	Concentration of chemical A & B	$\left(\frac{Mol}{L^{3}}\right)$
$a_{0}$	Uniform concentration of reactant A	$\left(\frac{Mol}{L^{3}}\right)$
$f,\;\overset{´}{g}$	Dimensionless concentration of chemical species A & B	$\left[-\right]$
$D_{A},D_{B}$	Mass diffusivity of species A & B	$\left[L^{2}T^{-1}\right]$
$K_{1}$	Homogeneous reaction constant for cubic autocatalysis	$\left({\left(\frac{Mol}{L^{3}}\right)}^{-2}T^{-1}\right)$
$K_{2}$	Reaction rate constant for the first order heterogeneous reaction	$\left[T^{-1}\right]$
$\beta_{T}$	Thermal expansion coefficient	$\left[K^{-1}\right]$
$\beta_{a}$	Concentration expansion coefficient	${\left(\frac{Mol}{L^{3}}\right)}^{-1}$
g	Acceleration due to gravity	$\left[LT^{-2}\right]$
$\bar{P}$	Dimensional pressure in the fixed frame	$\left[ML^{-1}T^{-2}\right]$
p	Dimensionless pressure $\left[-\right]$	$\left[-\right]$
${\bar{\zeta }}_{1},\;{\bar{\zeta }}_{2}$	Dimensional zeta potential of lower and upper walls	$\left[ML^{2}T^{-3}I^{-1}\right]$
$\zeta_{1},\;\zeta_{2}$	Dimensionless zeta potential of lower and upper walls $\left[-\right]$	$\left[-\right]$
$\bar{\phi }$	Dimensional electric potential	$\left[ML^{2}T^{-3}I^{-1}\right]$
ϕ	Dimensionless electric potential	$\left[-\right]$
z	Charge balance	$\left[-\right]$
e	Electric charge	$\left[IT\right]$
$T_{av}$	Mean temperature	$\left[K\right]$
$K_{B}$	Boltzmann constant	$\left[ML^{2}T^{-2}K^{-1}\right]$
${\bar{n}}_{\pm }$	Positive, negative ions	$\left[L^{-3}\right]$
$n_{0}$	Number density	$\left[L^{-3}\right]$
μ	Plastic viscosity	$\left[ML^{-1}T^{-1}\right]$
$\beta^{*}$	Eyring–Powell fluid constant	$\left[LT^{2}M^{-1}\right]$
$c^{*}$	Eyring–Powell fluid constant	$\left[T^{-1}\right]$
τ	Stress tensor	$\left[ML^{-1}T^{-2}\right]$
$B_{1},\;B_{2}$	Eyring-Powel fluid parameters	$\left[-\right]$
$E_{x}$	Electric field	$\left[MLT^{-3}I^{-1}\right]$
$B_{y}$	Magnetic field	$\left[MT^{-2}I^{-1}\right]$
$\lambda_{L}$	Debye length	$\left[L\right]$
$u_{HS}$	Helmholtz–Smoluchowski velocity	$\left[LT^{-1}\right]$
$U_{HS}$	Dimensionless Helmholtz–Smoluchowski velocity	$\left[-\right]$
ρ	Density of fluid	$\left[ML^{-3}\right]$
$\rho_{e}$	Net charge density	$\left[ITL^{-3}\right]$
ε	Relative permittivity of fluid	$\left[-\right]$
$\varepsilon_{0}$	Permittivity of free space	$\left[I^{2}T^{4}L^{-3}M^{-1}\right]$
k	Thermal conductivity	$\left[MLT^{-3}K^{-1}\right]$
δ	Wave number	$\left[-\right]$
$c_{p}$	Specific heat	$\left[L^{2}T^{-2}K^{-1}\right]$
$m_{e}$	Electroosmotic parameter	$\left[-\right]$
Pe	Peclet number	$\left[-\right]$
Re	Reynolds number	$\left[-\right]$
Pr	Prandtl number	$\left[-\right]$
Br	Brinkman number	$\left[-\right]$
γ	Joule heating parameter	$\left[-\right]$
Sc	Schmidt number	$\left[-\right]$
$G_{t}$	Temperature Grashoff number	$\left[-\right]$
$G_{a}$	Concentration Grashoff number	$\left[-\right]$
$M_{1}$	Homogeneous reaction parameter	$\left[-\right]$
$M_{2}$	Heterogeneous reaction parameter	$\left[-\right]$
